# High aspect ratio diamond nanosecond laser machining

**DOI:** 10.1007/s00339-023-06755-2

**Published:** 2023-06-15

**Authors:** Natalie C. Golota, David Preiss, Zachary P. Fredin, Prashant Patil, Daniel P. Banks, Salima Bahri, Robert G. Griffin, Neil Gershenfeld

**Affiliations:** 1 Department of Chemistry, Massachusetts Institute of Technology, Cambridge, MA 02139 USA; 2 Francis Bitter Magnet Laboratory, Massachusetts Institute of Technology, Cambridge, MA 02139 USA; 3 Center for Bits and Atoms, Massachusetts Institute of Technology, Cambridge, MA 02139 USA

**Keywords:** Laser ablation, Fundamentals of laser processing, Diamond laser machining, Nanosecond laser machining, Laser induced damage analysis

## Abstract

Laser processing of diamond has become an important technique for fabricating next generation microelectronic and quantum devices. However, the realization of low taper, high aspect ratio structures in diamond remains a challenge. We demonstrate the effects of pulse energy, pulse number and irradiation profile on the achievable aspect ratio with 532 nm nanosecond laser machining. Strong and gentle ablation regimes were observed using percussion hole drilling of type Ib HPHT diamond. Under percussion hole drilling a maximum aspect ratio of 22:1 was achieved with 10,000 pulses. To reach aspect ratios on average 40:1 and up to 66:1, rotary assisted drilling was employed using > 2 M pulse accumulations. We additionally demonstrate methods of obtaining 0.1° taper angles via ramped pulse energy machining in 10:1 aspect ratio tubes. Finally, effects of laser induced damage are studied using confocal Raman spectroscopy with observation of up to 36% increase in tensile strain following strong laser irradiation. However, we report that upon application of 600 °C heat treatment, induced strain is reduced by up to ~ 50% with considerable homogenization of observed strain.

**Supplementary Information:**

The online version contains supplementary material available at 10.1007/s00339-023-06755-2.

## Introduction

Diamond is a promising material for next generation quantum devices, micro-electromechanical systems (MEMS), biocompatible microfluidic and spectroscopic devices, and thermally optimized micro and power electronics [1–6]. Its superior material strength, thermal conductivity, high electrical resistivity, and highly transparent optical characteristics make it the ideal material for such applications. Decades of research and development have resulted in cost effective methods of producing synthetic single crystal diamond using either chemical vapor deposition (CVD) or high pressure high temperature (HPHT) methods [7, 8]. However, conventional machining processes are typically ineffective against the extreme hardness of diamond. For this reason, pulsed laser machining has become the standard for fabrication of diamond microstructures and devices.

During nanosecond laser machining, the incident irradiation is absorbed as thermal energy following thermal transfers and equilibration between electrons and the lattice within the laser pulse duration. In diamond, the absorbed thermal energy drives a phase transition of diamond to graphite and continued laser irradiation promotes the vaporization of graphite from the machined surface [9–12]. However, the ejection of graphite and other carbon-based vapors interferes with the laser beam, generating a plasma plume that considerably attenuates the incident irradiation, which is particularly detrimental to the energy intensive fabrication of high aspect ratio holes [9]. In contrast, when the pulse length is less than ~ 1 ps, ablation occurs through multi-photon absorption processes at wavelengths above 225 nm, which represents a threshold for the energy of a single photon to exceed the wide bandgap of diamond [10–15]. This mechanism offers the advantage of reduced heat affected zones and microcracking in the ablated material [13, 14]. Although ultrafast lasers offer the potential of reduced laser induced damage in diamond, nanosecond lasers are significantly more accessible with considerably lower cost/watt compared to ultrafast lasers.

While the body of literature supporting laser machining of diamond is extensive, there have been fewer investigations into achieving high aspect ratios in diamond at nanosecond time scales [16–24]. Aspect ratios between 12 and 15:1 are most commonly reported in diamond, with the highest achieved aspect ratio being 500:1 using a Bessel function focused beam and femtosecond laser [25–28].

In this paper we describe the achievable aspect ratio via percussion hole drilling and rotary assisted drilling. We characterize the effect of pulse energy and machining profile on the aspect ratio and taper, and report ~ 40:1 average aspect ratio holes in diamond. Finally, we characterize the induced strain as a measure of laser induced damage during nanosecond machining of 10:1 aspect ratio tubes. We observe a reduction in tensile strain following heat treatment at 600 °C for 24 h. Overall, we present a method of achieving high aspect ratios with a commercially available nanosecond laser machining system. This represents an accessible way of fabricating diamond structures for microelectronics, biomedical sensing, and nuclear magnetic resonance research [29].

## Materials and methods

### Diamond material

Type Ib HPHT diamond with four-point 100 crystal orientation was obtained from Element6 (Didcot, Oxfordshire, UK). For percussion hole drilling the material was 1.1 $\times$ 1.1 $\times$ 3 mm. For other samples, the material was 1.6 $\times$ 1.6 $\times$ 4 mm. The absorption coefficient and optical penetration depth of type 1b HPHT was determined using the same material, but with sample dimension of 10 $\times$ 10 $\times$ 1.1 mm.

### Laser machining system

Samples were fabricated using an Oxford Lasers (Didcot, Oxfordshire, UK) A-series laser micromachining system equipped with a Q-switched 532 nm diode-pumped Nd:YAG laser with a spot size of 13–14 μm. The pulse duration was ~ 20 ns at a repetition rate of 5 kHz with a maximum average power of 2.97 W. The laser power was attenuated using a computer controlled motorized optical attenuator and high contrast polarizer. Motion control in X and Y axes is achieved with a linear motor with a resolution of 0.25 μm and accuracy of ± 2 μm. Motion control along the beam propagation axis, Z, is achieved with a servomotor drive system that has a resolution of 0.5 μm and accuracy of ± 6 μm. Complete description of laser system is given in Fig. S1.

### Percussion hole drilling

Percussion hole drilling was performed with the diamond fixtured as shown in Fig. S2E, F with single pulse energies ranging from 6.8 to 594 µJ. The diameter and depth of percussion holes was determined using a Rigaku CT Lab HX130 (Toykyo, Japan) microCT scanner at 2.1 µm voxel size. Analysis was performed using ImageJ by compiling 3 adjacent radiographic slices and computing the average projection[30]. Hole diameters were then determined using a plotted profile from ImageJ and analyzed in MATLAB to determine the hole size measured at a constant gray-value to ensure uniformity of measurement.

### Rotary assisted drilling ~ 40:1 aspect ratio holes

An in-house fabricated precision rotary drilling apparatus was used with a Thorlabs (Newton, NJ, USA) DDR100 direct drive rotary stage to achieve a rotational frequency up to 2.76 Hz (166 RPM) during laser drilling, shown in Fig. S2. Focus, power, inner diameter, and feed rate were modulated using g-code written in Python and executed by Cimita control software. The high-aspect ratio holes were machined using a linear stage added in series with the rotary stage to translate the diamond stock while maintaining laser position relative to the center of rotation of the diamond material. The center of rotation was identified by taking a long exposure coaxial photograph, which allowed accurate positioning of the laser. During machining, initial radial passes at 250 µJ pulse energy traversed from the center of rotation of the diamond stock to the desired target radius at a feed rate of 10 µm/s and were repeated 10 times at both 0 and 250 μm of negative focus for a total of 140,000 equivalent pulses. In this case, negative focus is defined as a translation of the focal plane of the laser beam into the material. This was repeated with finishing passes of 400 µJ pulses, a feed rate of 5 μm/s and 25 µm depth increments of negative focus to a depth of 250 µm for a total of 1,540,000 equivalent pulses. During both machining cycles, the rotation rate was fixed at 66.6 RPM. The number of machining cycles investigated were 1, 2, and 3. Samples were characterized using a Rigaku CT Lab HX130 (Tokyo, Japan) with voxel sizes of 5.7 µm and a Hitachi FlexSEM1000 SEM (Tokyo, Japan) following sputter coating of 100 nm of gold onto the surface.

### Rotary assisted drilling of 10:1 aspect ratio tubes

Diamond rods were first turned to an outer diameter of roughly 520 μm using a single pulse energy of 296 µJ and a feed rate of 5 µm/s in lathe orientation shown in Fig. S2A, B. This was followed by a finishing pass at a feed rate of 1 µm/s at a single pulse energy of 236 µJ in which roughly 20 µm of material was removed from the diameter achieving average outer diameter of 500 µm with typical combined outer diameter eccentricity and taper of less than 6 µm over the 3 mm rod. Outer diameter measurements were performed using a Keyence (Osaka, Japan) TM-X5006 telecentric measurement system with ± 0.2 μm measurement positional accuracy. Inner diameters were machined by rotating the entire laser lathe apparatus, as shown in Fig. S2C, D with the laser machining axially into the rod. 224,000 equivalent rough-cut pulses were delivered using a radial feed rate of 50 μm/s with sample rotational speed of 166 RPM. Each radial pass was repeated 10 times, over 16 total negative focus increments of 250 μm, to a maximum depth of 4 mm. A finishing cycle delivered 2.24 M equivalent pulses using a feed rate of 50 µm/s and with focus negatively incremented by 25 µm to a maximum depth of 4 mm. Finally, the samples were re-orientated in lathe configuration and cross sectioned. The total machining time per inner diameter was ~ 90 min. Samples were characterized using a Hitachi FlexSEM1000 SEM (Tokyo, Japan) and taper was analyzed using SEM micrographs in Autodesk Inventor (San Rafael, CA, USA). 3-dimensional images of 10:1 aspect ratio tubes were acquired using a 20 × objective on a VK-X250 laser scanning confocal microscope (Osaka, Japan).

### Confocal Raman spectroscopy

Internal strain mapping was performed using a WITec alpha300 apryon Confocal Raman spectrometer (Ulm, Germany) at 532 nm using a 50 $\times$ object with a numerical aperture of 0.8 and irradiation power of 15 mW to avoid surface graphitization upon irradiation. Typical scan resolution was 0.5 µm in width and 1 µm in depth. The spectral resolution was maximized using an 1800 g/mm grating and the observed spectral stability and resolution of the ~ 1332 cm^−1^ diamond Raman shift was ± 0.01/cm. The linear relationship between Raman shift and strain was 360 MPa/cm, as previously determined [31, 32]. For each strain measurement, 3 locations were profiled, and 300 pixels were accumulated per location with an integration time of 0.2 s. Each reported strain is the average of these 3 locations for a total of 900 pixel accumulations. No significant differences were observed in the Raman spectra as a function of location down the cross sectioned tube. Between each sample strain measurement, the Raman shift of the spectrometer internal diamond standard sample was used to account for any spectrometer instabilities. The reference samples were unmodified stock material corresponding to each machined sample shown in Fig. 3. Highly ordered pyrolytic graphite (HOPG) ZYB quality was purchased and used without modification from Ted Pella (Redding, CA, USA) as a graphite standard. The Raman shift distribution in the sample was determined using a Lorentzian fit in WITec Project software. Samples were then subjected to 24 h of heat treatment at 600 °C in air and atmospheric pressure (~ 100 kPa) in a tabletop oven. After 24 h the samples were allowed to cool to room temperature over ~ 2–4-h duration. Sample mass was taken before and after heat treatment. The Raman measurements were then repeated as described above.

## Results and discussion

### Percussion hole drilling

**Fig. 1:**
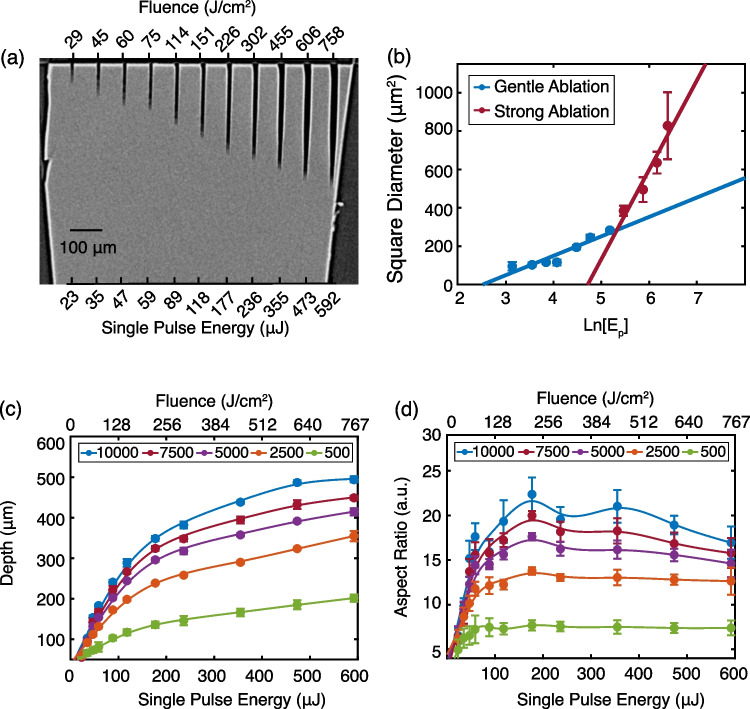
Percussion hole drilling. **a** MicroCT radiograph showing the effect of pulse energy and fluence on the diameter and depth of the ablated percussion hole. **b** Distinct gentle and strong ablation regimes were observed during percussion hole drilling, shown for 5,000 pulses. **c** The achievable depth as a function of single pulse energy or fluence and number of laser pulses.** d** The aspect ratio was calculated using the depth and percussion hole diameter as a function of laser pulses and single pulse energy or fluence. In (**c**,** d**)  the top panel indicates the number of incident pulses which was varied from 10,000 to 500 pulses

For a given number of pulses, increasing the pulse energy increased the observed hole depth and diameter, as shown in Figs. 1 and S8. The ablation thresholds for multiple pulse numbers were determined by the linear relation in Eq. (1):

1 $$D^{2} = 2 \omega_{0}^{2} \ln \frac{{E_{{\text{p}}} }}{{E_{{{\text{TH}}}} }}$$

We observed distinct strong and gentle ablation regimes in HPHT diamond, as shown in Figs. 1b and S9. While two distinct ablation regimes are most observed during ultrafast machining processes; they have been observed with nanosecond machining, including in diamond [21, 33–35]. Gentle ablation occurs when the single pulse energy is below ~ 125 µJ and in this regime the spot size of the laser was determined to be 14.1 ± 0.8 µm. The gentle ablation threshold was determined to be subject to incubation effects, with an incubation coefficient of 0.919 ± 0.008 as determined by Eq. (2), where N is the number of pulses, ${\Phi }_{N}$ is the multiple pulse ablation threshold, and ${\Phi }_{1}$ is the single pulse ablation threshold.

2 $$\ln \left[ {N \cdot {\Phi }_{N} } \right] = S\ln \left[ N \right] + \ln \left[ {{\Phi }_{1} } \right].$$

An incubation coefficient < 1 indicates that the ablation threshold decreases with multiple pulses due to accumulation of laser induced damage regions that more strongly absorb laser irradiation. Incubation effects are expected in diamond, given the starting material is largely transparent with initial absorption driven only by crystal inclusions and defects.

Accordingly, for 10,000 pulses the ablation threshold fluence was 12.4 ± 1.4 J/cm^2^ which increased to 14.9 ± 3.5 J/cm^2^ for 500 pulses, and a single pulse threshold fluence was determined to be 29.5 ± 1.3 J/cm^2^. The multiple pulse ablation thresholds are within literature values for nanosecond machining of single crystal diamond, particularly for HPHT diamond which has a higher ablation threshold than CVD diamond [21, 36, 37]. The low absorption coefficient of HPHT diamond and thermodynamic stability of diamond contribute to the large threshold fluences.

The strong ablation regime occurs at fluences many times the threshold fluence, in this work the strong ablation regime began between 127 and 162 J/cm^2^ and without the observation of incubation effects. In this regime, we observed 3–7 µm eccentricities in hole diameter between the x and y radiograph cross sections, which resulted in variability in average hole size. The decrease in circularity of percussion holes at higher pulse energies has been observed previously [38]. The higher energy pulses may result in smaller graphite particulate with higher ejection velocities, resulting in a more rapid expulsion of graphite leading to a larger relative increase in hole diameter, evidenced by the steeper observed slope in Figs. 1b and S9.

The maximum depth of percussion hole drilling achieved was 493.6 ± 6.4 µm using a single pulse energy of 592 µJ and 10,000 laser pulses. Within the gentle ablation regime, the hole depth increased rapidly with single pulse energy for a given number of laser pulses, Fig. 1c. However, in the strong ablation regime, the additional depth provided by higher energy pulses was reduced in magnitude relative to the gentle ablation regime.

The maximum achievable aspect ratio was 22.4 ± 1.8 using 10,000 pulses at a single pulse energy of 177 µJ and is shown in Fig. 1d. At higher powers, the increase in hole diameter dominated the nominal gain in depth resulting in lower aspect ratios when > 5000 pulses were used. As the pulse number was decreased, the aspect ratio plateaued at ~ 12 and 7 for 2500 and 500 pulses at pulse energies above ~ 75 µJ, respectively. We note the oscillatory nature of the aspect ratio curve between 150 and 350 μJ single pulse energy to be most likely an experimental artifact. When measured with a power meter, the laser energy was found to be constant, as shown in Fig. S3. The maximum aspect ratios are obtained below the highest pulse energies applied, while maximum depths are achieved at highest pulse energies and pulse accumulations. Overall, the results of percussion hole drilling demonstrate distinct gentle and strong ablation regimes and served as a basis for understanding ablation dynamics in HPHT diamond.

### Rotary assisted drilling of ~ 40:1 aspect ratio holes

Higher aspect ratios of up to 66:1 were achieved using a rotary assisted drilling while the beam focus was negatively incremented into the workpiece, as shown in Fig. S2E, F. Large equivalent pulse numbers were used to achieve the high aspect ratio holes, with a total of 1.68 M pulses per machining cycle. The equivalent pulse number was computed as $2{w}_{0}f/v$, where *f* is the laser repetition rate and *v* is the traverse rate. The average aspect ratio achieved was 42.5, 38.6 and 39.3, respectively, where the number of machining cycles was increased from 1 to 3. The largely unchanging aspect ratio with number of machining cycle repetitions indicates pulse saturation was achieved. The formation of a chamfer feature at the entrance hole resulted in minimum aspect ratios of 20.1, 20.5, and 20.1, with chamfer geometry dependent on negative focus depth, Fig. S12A, B. We define chamfer as a 100–200 µm deep feature with a sloped edge at an angle roughly one order of magnitude greater than the overall internal taper angle of the hole. On cross sectional radiographs and micrographs, the chamfered region is distinct from the inner diameter taper extending throughout the length of the hole and is shown in Fig. S14.

**Fig. 2:**
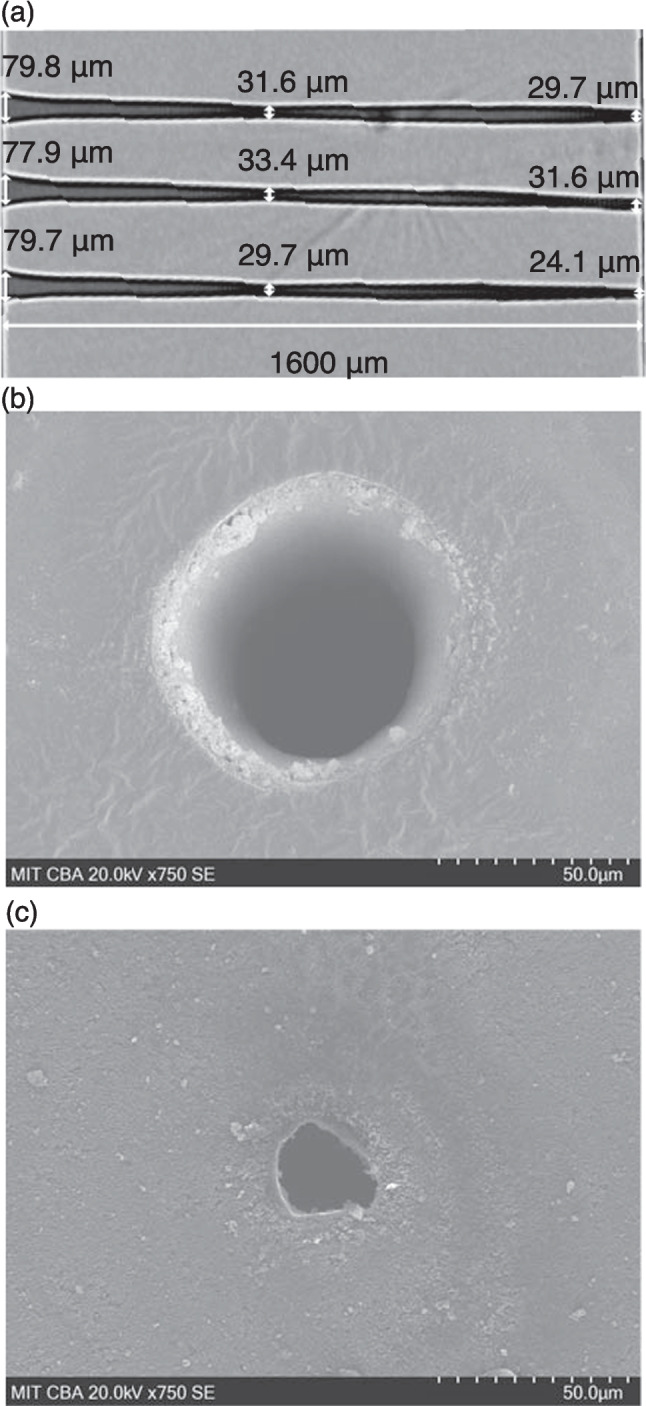
Average 40:1 aspect ratio holes in diamond (**a**). MicroCT cross section showing three holes fabricated with rotary assisted drilled to a negative focus of 250 µm through 1.6 mm of HPHT diamond. From bottom to top the number of machining cycles was increased from 1 to 3. **b** The SEM of the entrance hole shows evidence of chamfer at the top of the hole. **c** SEM of exit of the bottom hole, shows the expulsion pattern, reduced circularity, and evidence of internal taper

The exit hole was undersized, with corresponding aspect ratios of 66.4, 50.6 and 53.9 for 1–3 machining cycles. The non-uniform hole geometry and reduction in exit hole circularity, shown in Fig. 2c, is related to the complex dynamics of material vaporization, melt and debris expulsion. As machining progresses, the absorptive losses increase within vaporized graphite and plasma, reducing the effective energy density and ablation efficiency resulting in the hourglass shape of the hole. Finally, occlusion of the diverging laser beam at depths far exceeding the Rayleigh length of the laser beam results in the formation of chamfer and overall taper of ~ 0.9°. The far field divergence angle of the gaussian beam is 1.38°. Overall, we demonstrate use of high accumulated pulse numbers to achieve, to our knowledge, the highest aspect ratios in diamond using nanosecond laser machining to date.

### Rotary assisted drilling of 10:1 aspect ratio tubes

**Fig. 3:**
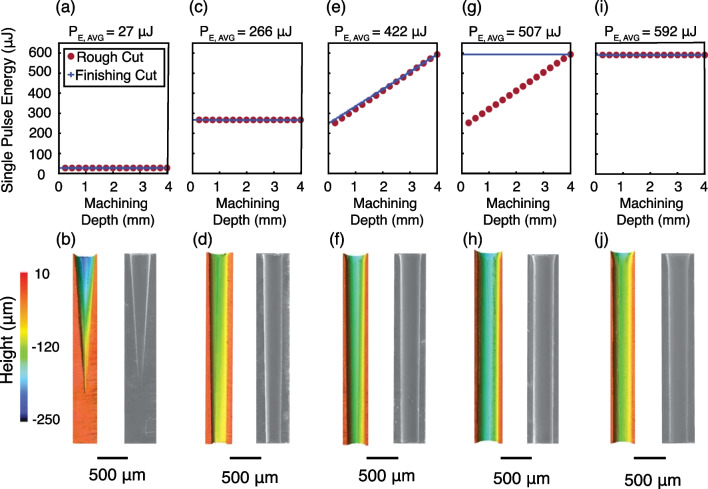
Machining 10:1 aspect ratio tubes. Top panels show the machining profile used. The single pulse energy was either held constant while the machining focus was negatively incremented down the machining depth (**a**–**d**,** i**,** j**) or ramped (**e**-**h**) during initial rough cuts or final finishing cuts. The bottom panels show the laser confocal scanning microscope images of the inner surface of a cross section of the 10:1 aspect ratio tube. SEM images are provided to show the entire structure, uncropped images provided in Supplementary information

**Fig. 4:**
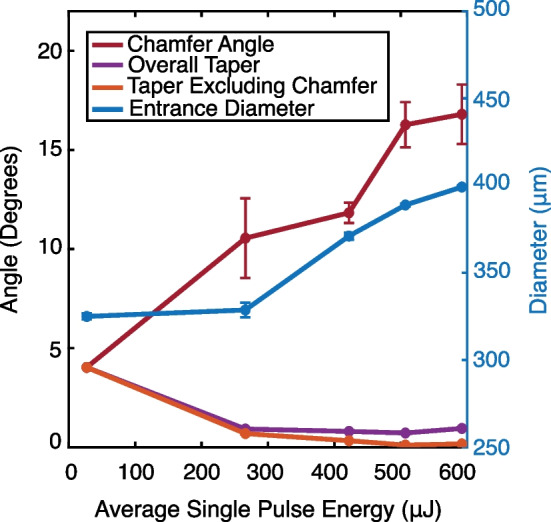
Characteristics of 10:1 aspect ratio tubes. The effect of average single pulse energy on the chamfer angle, overall taper, taper excluding chamfer and the entrance diameter is determined. At high average single pulse energies, taper can be reduced, however, up to ~ 17° of chamfer occurs, corresponding to nearly a 100 μm increase in the entrance hole diameter

While aspect ratios up to 66.4:1 were achieved, the inner diameter was non-uniform, tapered, and with poor circularity. While micro-hole arrays have potential uses in microfluidics and for thermal management, the fabrication of micro-tubes has additional applications in biocompatible devices and for magnetic resonance sample holders [39]. For the latter application, diamond sample holding tubes are pneumatically driven to rotational rates exceeding 7 $\times$ 10^6^ RPM [29, 39]. As such, the inner diameter of the hole must be uniform, with near zero internal taper, and with highly concentric inner and outer diameters (e.g., < 5–10 µm concentricity errors). We aimed to minimize internal taper angle by varying the irradiation profile for a constant target aspect ratio of 10:1, as shown in Fig. 3 and described previously. The impact of the machining profiles on the chamfer angle, overall taper, taper excluding chamfer, and entrance diameter is provided in Fig. 4. Three samples were machined with a constant single pulse energy (Fig. 3a, c, i). A constant single pulse energy of 27 µJ, nearly 3 times the gentle ablation threshold, was insufficient to promote thermal ablation throughout the entire 3 mm thick diamond, with a resulting ~ 4° internal taper along the maximum depth machined of ~ 2.1 mm, as shown in Fig. 3a, b. At all higher single pulse energies, the inner diameter was machined throughout the entire 3 mm sample. In all samples, the single pulse energy was linearly correlated with the chamfer angle and the entrance diameter. Maximum chamfer angles up to 16.8° and entrance diameters ~ 100 µm greater than the target diameter were observed with constant, high-energy finishing passes at 592 µJ. The length of the chamfer was roughly 100–200 µm and is formed by the diverging beam’s expanded beam waist having sufficient energy to induce ablation.

While chamfer was positively correlated with pulse energy, minimization of taper angle was generally achieved with high average pulse energies, as shown in Fig. 4. The lowest overall taper angles of 0.80° and 0.72° were achieved using ramped pulse energy machining with average pulse energies of 422 or 507 μJ, shown in Fig. 3e, f and g,h, respectively. However, at a pulse energy of 592 μJ, roughly 5 × the strong ablation threshold, the overall taper increased to 0.95°, comparable to the sample machined with a constant pulse energy of 226 μJ or that of the 40:1 aspect ratio holes. At such pulse energy, the constant application of laser irradiation with a beam waist of up to 55 µm containing sufficient energy density to generate ablation results in the observed chamfer formation that dominates the overall taper of the hole.

In cases where the 10°–20° chamfer angle is detrimental, the 100–200 μm deep chamfer can simply be parted from the material following inner diameter machining. As such, the inner diameter taper angle excluding chamfer represents the smallest fundamental taper angle achievable. In the present study this was minimized using high energy finishing passes with a minimum internal taper angle excluding chamfer between 0.11 and 0.17°, notably below the beam divergence angle. We conclude the effect of high pulse energy is on the dynamics of debris and vapor ejection from the machined hole. When the energy density is ramped toward higher fluence at the base of the hole, a greater fraction of ablated material is removed from the bottom of the hole and reduces beam attenuation, as reported in the literature [40]. In contrast, at lower pulse energies the upward material removal fraction is dominant, with debris, vapor, and plasma strongly attenuating the incident irradiation. Finally, the use of high-power pulses may further reduce particulate size, resulting in higher ejection velocities and reduced taper.

### Confocal Raman spectroscopy

While use of high pulse energies aided in the fabrication of low internal taper 10:1 aspect ratio tubes, high energy nanosecond laser machining can result in large heat affected zones with associated laser induced damage. Confocal Raman spectroscopy is a commonly used method of mapping the internal strain in diamond, in addition to characterizing the effects of laser machining [9, 20, 31, 32, 41–43]. In this study, confocal Raman spectroscopy was applied to characterize the effects of laser machining including the formation of sp^2^ carbon and generation of laser induced strain. The ablation mechanism was confirmed by the presence of surface localized graphite, in addition to generation of amorphous carbon, shown in Fig. S15. From Raman images, the laser induced surface graphite layer was found to be 1–2 µm thick.

**Fig. 5:**
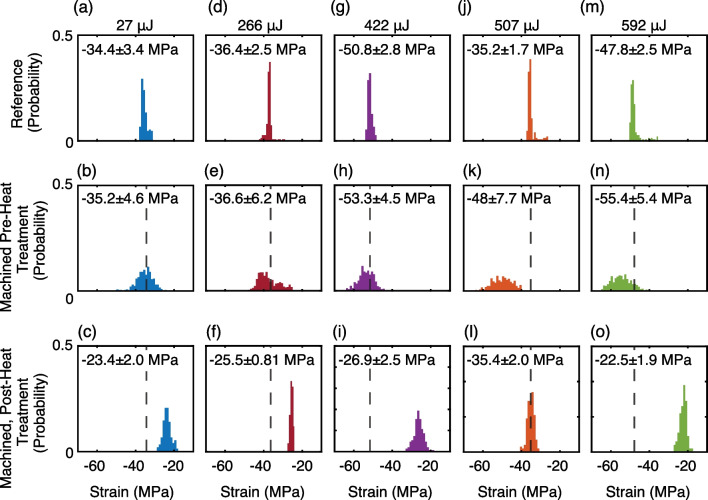
Laser induced strain in single crystal diamond. The effect of laser machining on internal strain in the diamond crystal was determined using confocal Raman spectroscopy. In the top row (**a**,** d**,** g**,** j**,** m**), reference sections from the 10:1 aspect ratio tube and unmachined by the Oxford laser were used as a standard sample. All standard samples showed tensile strains between ~ 35–51 MPa in magnitude with narrow strain distributions within a sample. In the middle row  (**b**, **e**,** h**,** k**,** n**) samples were studied through the first 15 μm of material and the distribution of internal strain computed. The dashed line corresponds to the average strain in the reference sample. The bottom row (**c**,** f**,** i**,** l**,** o**) shows the same machined samples following 24 h of heat treatment at 600 °C. In all cases the strain induced by laser machining is reduced and becomes comparable or reduced to that in the reference sample

Raman strain mapping was performed on each of the machined cross sections shown in Fig. 3, as well as unmachined samples of the stock material. The stock material had been previously laser cut into trapezoidal logs, and Raman mapping was performed on the first 15 μm of unmachined surface. The linear relationship between Raman shift and strain was 360 MPa/cm^−1^, as previously determined [31, 32]. As shown in Fig. 5, reference samples without in-house machining had tensile strains ranging from -34 to -51 MPa. In these samples, areas of lowest strain were found within the first 1–2 μm of the surface, depicted in Fig. S15. Further below the surface, greater magnitudes of tensile strain were observed, but was homogeneously distributed. The observed tensile strain indicates an increase in effective bond length relative to the ideal diamond crystal lattice. The baseline strain is attributed to two factors: (1) the presence of defects such as P1 center inclusions within the stock material and (2) prior exposure to unknown levels of laser irradiation during stock material processing by Element6.

In all machined samples, laser ablation resulted in a broadened strain distribution, particularly within the first 2.5 µm of the surface relative to reference samples. For samples machined with average single pulse energies of 27, 266 and 422 µJ, less than a 5% net increase in strain was observed. This represents an increase in tensile strain of ~ 3 MPa, which is on the order of the stability of the spectrometer across measurements. However, when 592 µJ finishing passes with ~ 2.2 M equivalent laser pulses were used, we observed a resultant 16–36% increase in induced strain. This corresponds to a total tensile strain of 48 and 55.4 MPa. In these two samples, the accumulated exposure to high energy laser irradiation and prolonged thermal ablation resulted in additional elongation of the carbon–carbon bonds in the crystal lattice.

Following laser machining, samples were subjected to oxidative heat treatment at atmospheric pressure for 24 h to remove graphite from the surface of the material. At temperatures above 700 °C and ambient pressure, diamond can begin to burn and form graphite, as such, the temperature was maintained at 600 °C [44–46]. Representative samples were weighed before and after heat treatment with typical mass changes on the order of ± 2%, representing insignificant changes in diamond crystal volume. Raman spectroscopy was repeated and in all cases a reduction in total strain was observed, including in reference samples. Typical reduction in strain for reference samples following heat treatment was ~ 32%. A 26–59% percent reduction of strain in machined samples following heat treatment was uncorrelated with the machining power applied. The laser induced broadening of strain distributions was observed to be reversible, as evidenced by the comparable standard deviation in strain measurement for all samples following heat treatment, provided in Fig. 5 and Table 1. For additional Raman images and detailed strain changes in all samples, we refer the reader to the supporting information.

**Table 1:** Measured strain values from Confocal Raman spectroscopy

Average single pulse energy (µJ)	Reference, pre-heat treatment strain (MPa)	Reference, post heat treatment strain (MPa)	Reference sample change in strain heat treatment	Machined sample, pre-heat treatment strain (MPa)	Laser induced strain (%)	Machined sample, post heat treatment strain (MPa)	Sample change in strain heat treatment
27	– 34.4 ± 3.4	– 20.9 ± 3.2	– 39%	– 35.2 ± 4.6	2%	– 23.4 ± 2.0	– 34%
266	– 36.4 ± 2.5	– 29.9 ± 1.8	– 18%	– 36.6 ± 6.2	0.5%	– 25.5 ± 0.81	– 30%
422	– 50.8 ± 2.8	– 32.6 ± 2.4	– 36%	– 53.3 ± 4.5	5%	– 26.9 ± 2.6	– 50%
507	– 35.2 ± 1.7	– 31.7 ± 2.0	– 10%	– 48.0 ± 7.7	36%	– 35.4 ± 2.0	– 26%
592	– 47.8 ± 2.5	– 20.4 ± 2.7	– 57%	– 55.4 ± 5.0	16%	– 22.5 ± 1.9	– 59%

The reference sample strain measurements are provided before and after heat treatment, all samples show at least a ~ 10% reduction in strain and up to 57% reduction. The net laser induced strain as a percent increase is provided prior to heat treatment. Following heat treatment, the strain was re-measured in the machined samples and the percent reduction in strain is provided, relative to the machined sample prior to heat treatment

High temperature (> 1700 °C) annealing using either high pressure (> 50 kBar) or low pressures (< 0.4 Bar) in diamond has been previously found to reduce strain and modify defects such as nitrogen vacancy centers in diamond [47–51]. In these cases, strain was relieved via vacancy diffusion and crystal lattice defect rearrangement that reduces non equilibrium bond distances within the lattice. In the case of heat treatment up to 800° C in nanodiamonds, strain release has been observed, however, this was concurrent with crystal volume loss and surface graphitization via surface etching [52]. A twofold mechanism was proposed in which the strained, surface exposed crystal was graphitized, and the interior lattice structure was homogenized by defect annihilation. In a recent study, low temperature annealing up to 500 °C reported reduction in radiation stains of natural diamond via reduction in interstitial carbon vacancy complexes at lower activation energies than nitrogen vacancies [53].

In the present study, we did not observe heat treatment induced graphitization of any samples or crystal volume loss. We hypothesize the thermal energy supplied was sufficient to aid in the dispersal of unstable impurities, such as metal catalyst inclusions, that led to more homogeneous and slightly decreased strains, of low initial magnitude. In HPHT/LPHT annealing, changes in strain are often on the order of a few gigapascals, indicating much larger changes to the diamond crystal lattice. While Raman spectra show no evidence of graphitization, we cannot fully exclude graphitization of the surface and thermal etching as the mechanism of strain reduction. Further study should also include electron paramagnetic resonance (EPR) studies to determine if charge transfer or transformation of defect states derived from P1 center (C-center) substitutions has occurred.

## Conclusions

In conclusion, we have characterized the effect of pulse energy, pulse accumulation, machining depth, and drilling method on nanosecond laser machining of diamond. We observed two distinct machining regimes in HPHT diamond, dictated by the thermal diffusivity of the diamond in relation to accumulated thermal energy within the lattice. The achievable aspect ratio is dictated by the pulse energy and number, with a required balance between excessive chamfer formation at high pulse energy and machining depth. At maximum, 66:1 aspect ratio was obtained, with the average aspect ratio of ~ 40:1 being achieved following saturation of multiple pulse (> 10^6^) effects in diamond. While low internal taper is achieved with high pulse energy, the concurrent formation of chamfer must be carefully controlled. A minimum 0.11° internal taper angle was achieved over ~ 3 mm lengths at an aspect ratio of 10:1, representing an important step toward high precision, low taper structures in diamond. Finally, the nanosecond diamond laser machining process was profiled using confocal Raman spectroscopy and formation of surface localized amorphous carbon and graphite was confirmed. All samples had between -30 to -55 MPa of tensile strain, which was increased by ~ 36% upon application of high energy laser irradiation. In all cases, the distribution of internal tensile strains was broadened particularly within the first 2.5 µm of diamond following laser irradiation. However, these effects were reduced following heat treatment at 600 °C. Overall, the results presented here demonstrate the utility of nanosecond machining in the fabrication of high aspect ratio diamond structures and a method of mitigating nanosecond laser induced changes in diamond.

## Supplementary Information

Below is the link to the electronic supplementary material.

Supplementary file1 (DOCX 5544 KB)

## Data Availability

The datasets generated during and/or analyzed during the current study are available from the corresponding author upon request.
