# Supplementary material for: High aspect ratio diamond nanosecond laser machining
Source: Appl Phys A Mater Sci Process. 2023 Jun 15;129(7):490. doi: 10.1007/s00339-023-06755-2 (PMC10271884; doi:10.1007/s00339-023-06755-2)

**Supplementary Materials for:**

**High Aspect Ratio Diamond Nanosecond Laser Machining**

Natalie C. Golota^1,2,*^ †, David Preiss^3^ †, Zachary P. Fredin^3^ , Prashant Patil^3^, Daniel P. Banks^1,2^, Salima Bahri^1,2^, Robert G. Griffin^1,2^, Neil Gershenfeld^3^

**Affiliations**

^1^ Department of Chemistry, Massachusetts Institute of Technology, Cambridge, MA, 02139, USA.

^2^ Francis Bitter Magnet Laboratory, Massachusetts Institute of Technology, Cambridge, MA, 02139, USA

^3^ Center for Bits and Atoms, Massachusetts Institute of Technology, Cambridge, MA, 02139, USA

* Corresponding author. Email: ngolota@mit.edu

† These authors contributed equally to this work.

This PDF file includes:

**Figures**

[Figure S1. Laser system block diagram. 3](#_Toc136355326)

[Figure S2. Homebuilt laser-lathe apparatus. 4](#_Toc136355327)

[Figure S3. Laser power output. 5](#_Toc136355328)

[Figure S4. Optical properties of Type 1b HPHT Diamond. 6](#_Toc136355329)

[Figure S5. SEM of percussion holes. 7](#_Toc136355330)

[Figure S6. MicroCT Cross section views of percussion holes in the y-direction. 8](#_Toc136355331)

[Figure S7. MicroCT cross section views of percussion holes in the x-direction. 9](#_Toc136355332)

[Figure S8. Inner diameter of percussion holes. 10](#_Toc136355333)

[Figure S9. Determination of multiple pulse ablation threshold. 12](#_Toc136355334)

[Figure S10. Single pulse ablation threshold. 13](#_Toc136355335)

[Figure S11. Entrance and Exit holes of ultra-high aspect ratio holes. 14](#_Toc136355336)

[Figure S12. Chamfer as a function of machining depth. 16](#_Toc136355337)

[Figure S13. Uncropped SEMs of 10:1 Aspect Ratio tubes. 17](#_Toc136355338)

[Figure S14. Definition of chamfer and taper angles. 18](#_Toc136355339)

[Figure S15. Surface Graphitization during ns laser machining. 19](#_Toc136355340)

[Figure S16. Raman images of 10:1 aspect ratio tube cross sectional samples. 20](#_Toc136355341)

[Figure S17. Pre and Post Heat Treatment Strain. 21](#_Toc136355342)

**Tables**

[Table S1. Ablation threshold of HPHT diamond. 11](#_Toc136358167)

[Table S2. Inner diameters of 40:1 aspect ratio holes. 15](#_Toc136358168)

[Table S3. Aspect Ratio of ~40:1 holes. 15](#_Toc136358169)

[Table S4. Chamfer, taper, and diameter of 10:1 aspect ratio tubes. 18](#_Toc136358170)

Section 1. Laser machining system characterization


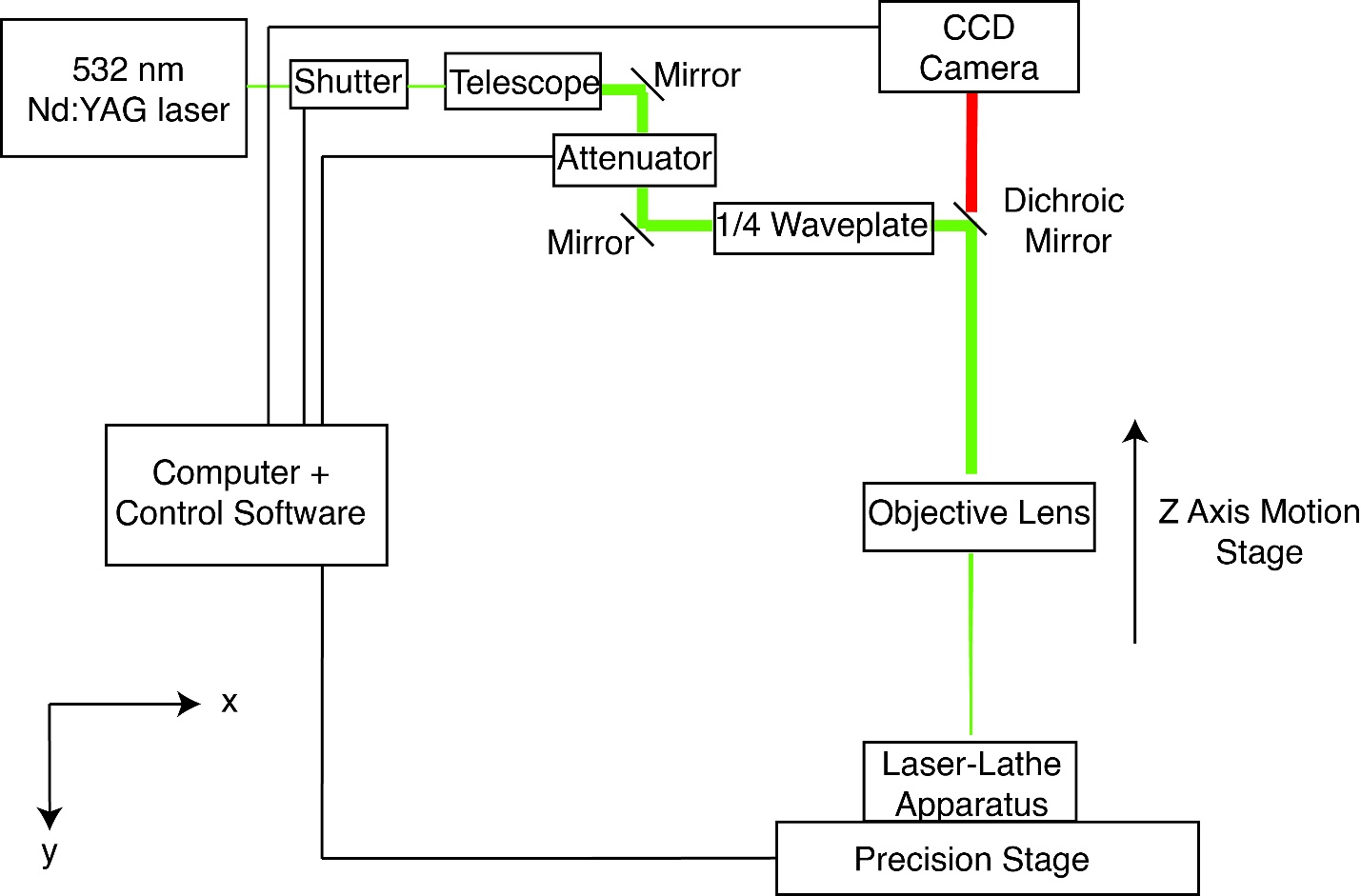


Figure S1. Laser system block diagram.

A 532 nm Q-switch diode-pumped-solid-state Nd:YAG laser operating at a maximum average power of 3 W with a 5 kHz repetition rate was used. The laser beam is expanded to 10 mm prior to the objective lens which focuses the beam to a 10 µm spot size over a focal length of 100 mm. The focus of the beam incident onto a machining part is controlled through a precision servomotor drive system with a linear motor with resolution of 0.25 μm and accuracy of ±2 μm. The workpiece is fixtured onto a homebuilt laser-lathe shown in Figure S2 and is manipulated in the x,y directions using a high precision stage. Finally, an on-axis CCD camera equipped with a laser-cross hair alignment aid is used to confirm workpiece alignment, laser position and machining progress.


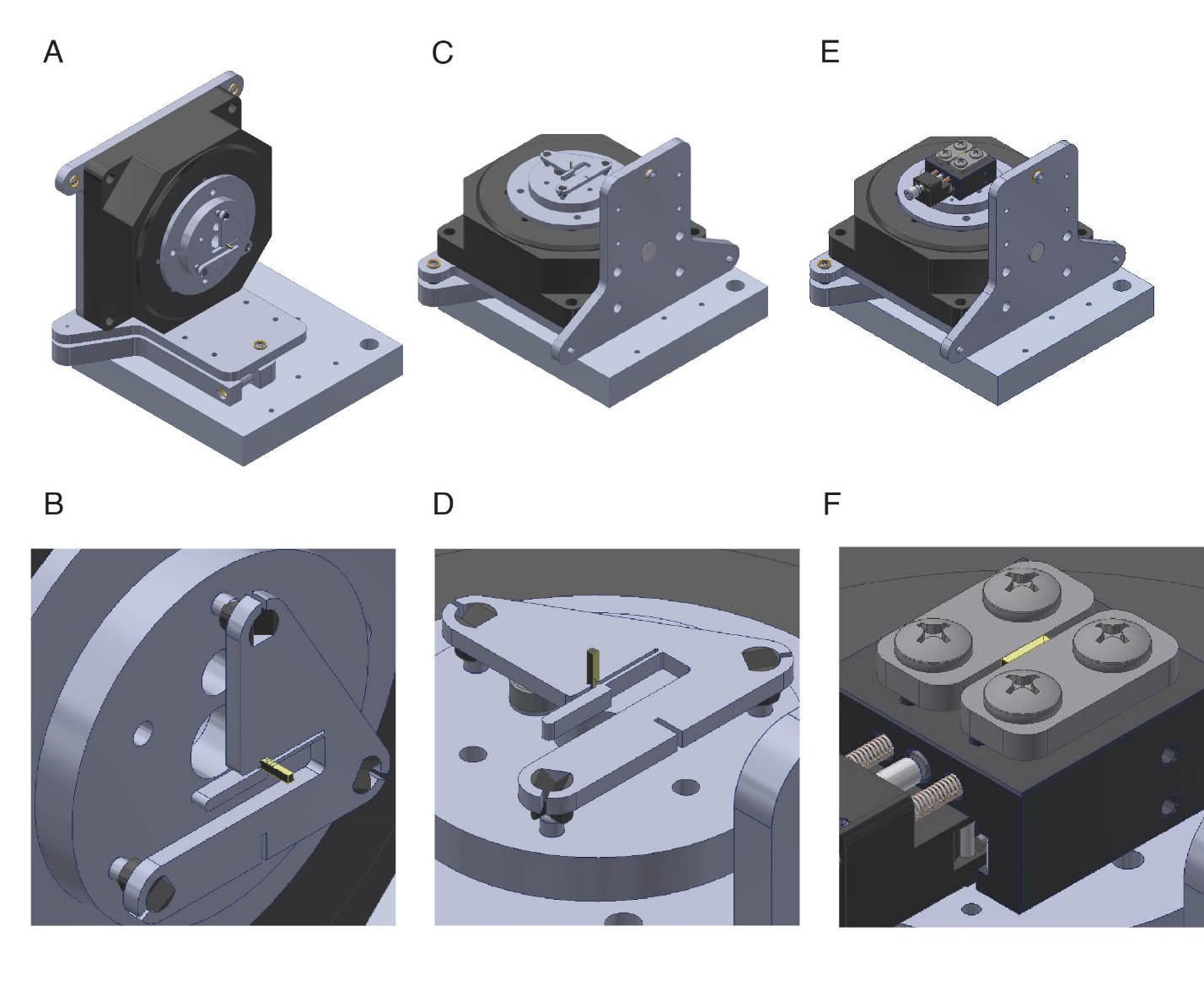
**Figure S2. Homebuilt laser-lathe apparatus.**

In all configurations, the laser beam is incident from the top of the image. Baseplates of laser lathe allow for adjustment of pitch and yaw in addition to kinematic couplings for reliable re-orientation between machining schemes. The rotary stage provides 1000 degrees/s rotational speeds used during machining inner and outer diameters of high aspect ratio holes. (A,B). Laser lathe orientation for outer diameter machining, used to turn the 10:1 aspect ratio tubes. (C,D). Orientation used for machining the inner diameter of 10:1 aspect ratio tubes. (E,F). Fixture used to machine ~40:1 aspect ratio tubes.


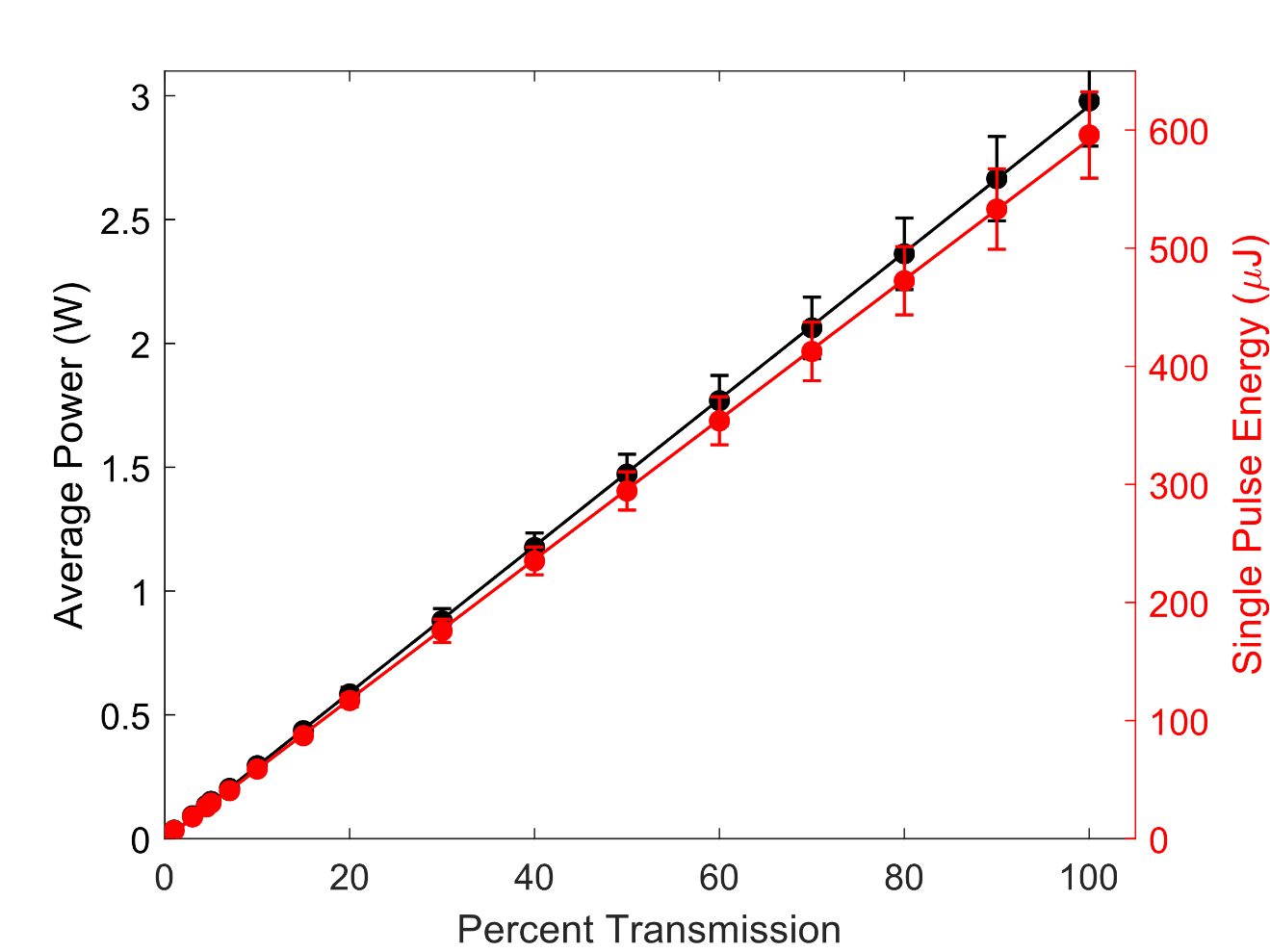


Figure S3. Laser power output.

The measured average laser power as a function of percent transmission was measured using the built in power meter. A linear relation is maintained between the transmitted laser power and pulse energy across the powers used in this study.


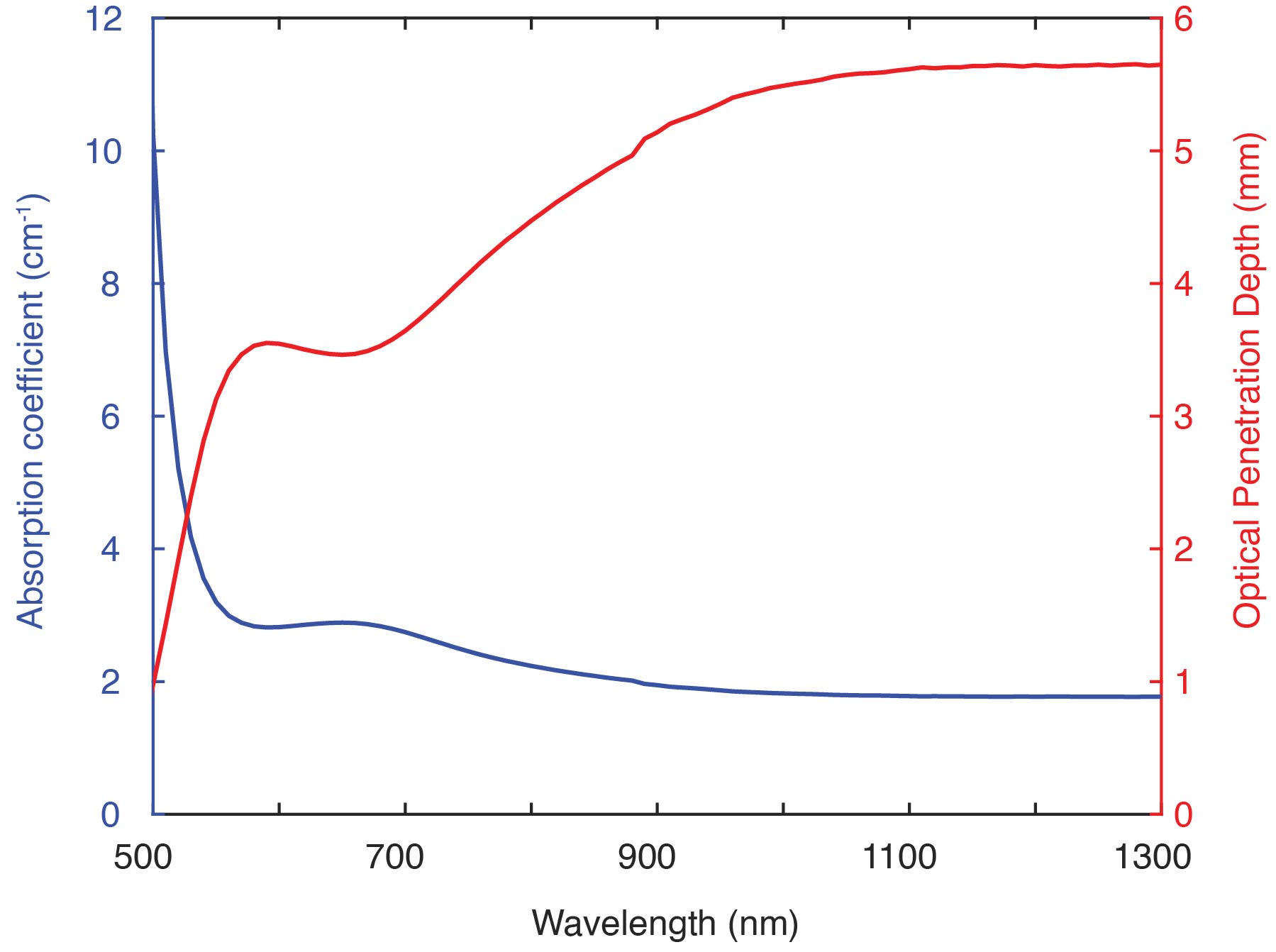


­­Figure S4. Optical properties of Type 1b HPHT Diamond.

Absorption coefficient and optical penetration depth for Type 1b HPHT diamond. Measured was performed using a Perkin Elmer Lambda 1050 UV/VIS spectrophotometer on a 1.1 mm thick diamond sample. At 532 nm, the absorption coefficient was found to be 4.19 ± 0.002 cm^-1^. The optical penetration depth was calculated to be 2.39 ± 0.13 mm at 532 nm.

**Section S2. Ablation threshold determination**


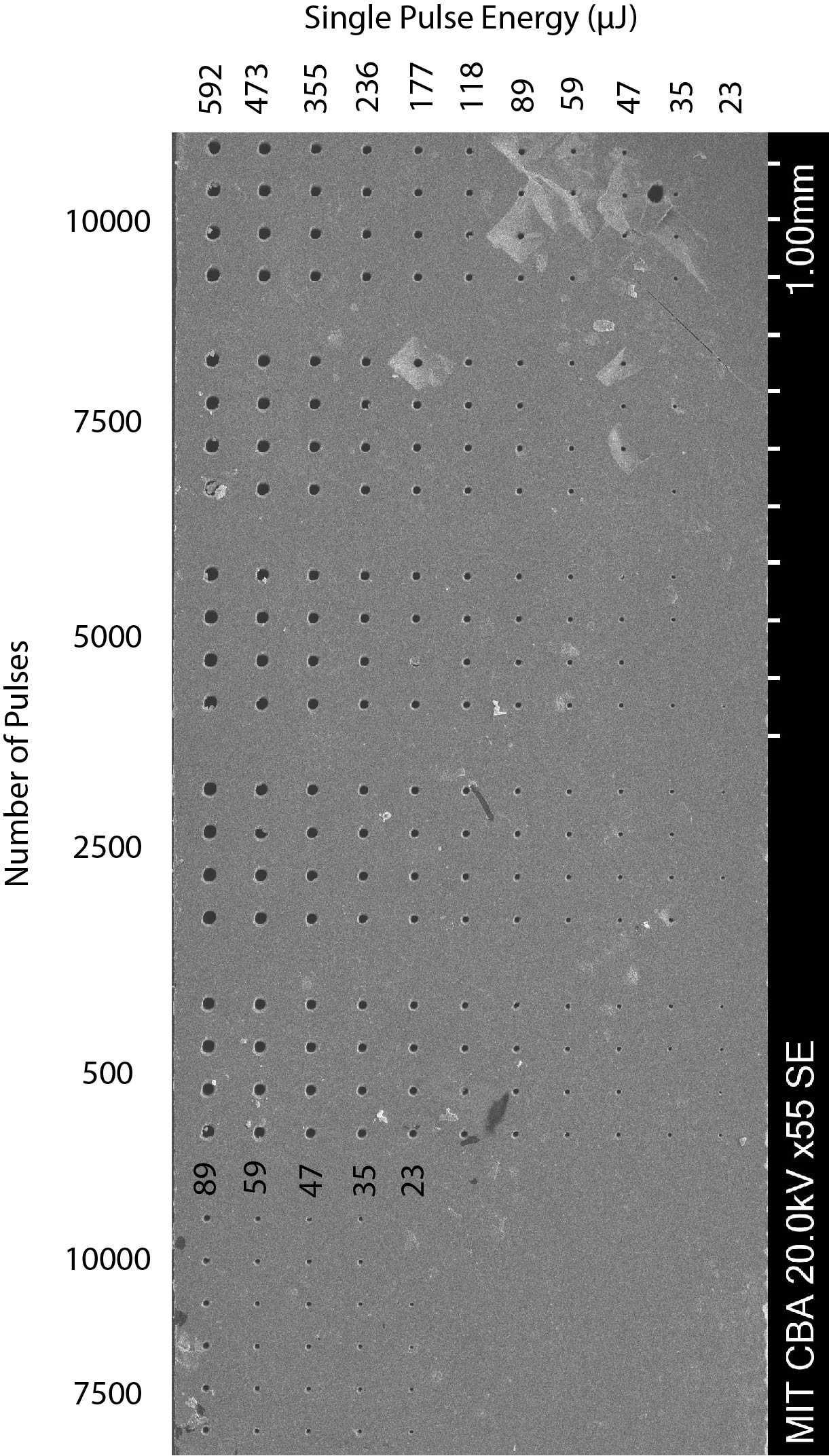


Figure S5. SEM of percussion holes.

SEM of percussion holes used to determine ablation threshold. Extensive cracking was observed at the 5 lowest powers for 7500 and 10000 pulses. These tests were repeated and provided in the left most section of the figure. Holes were repeated four times.


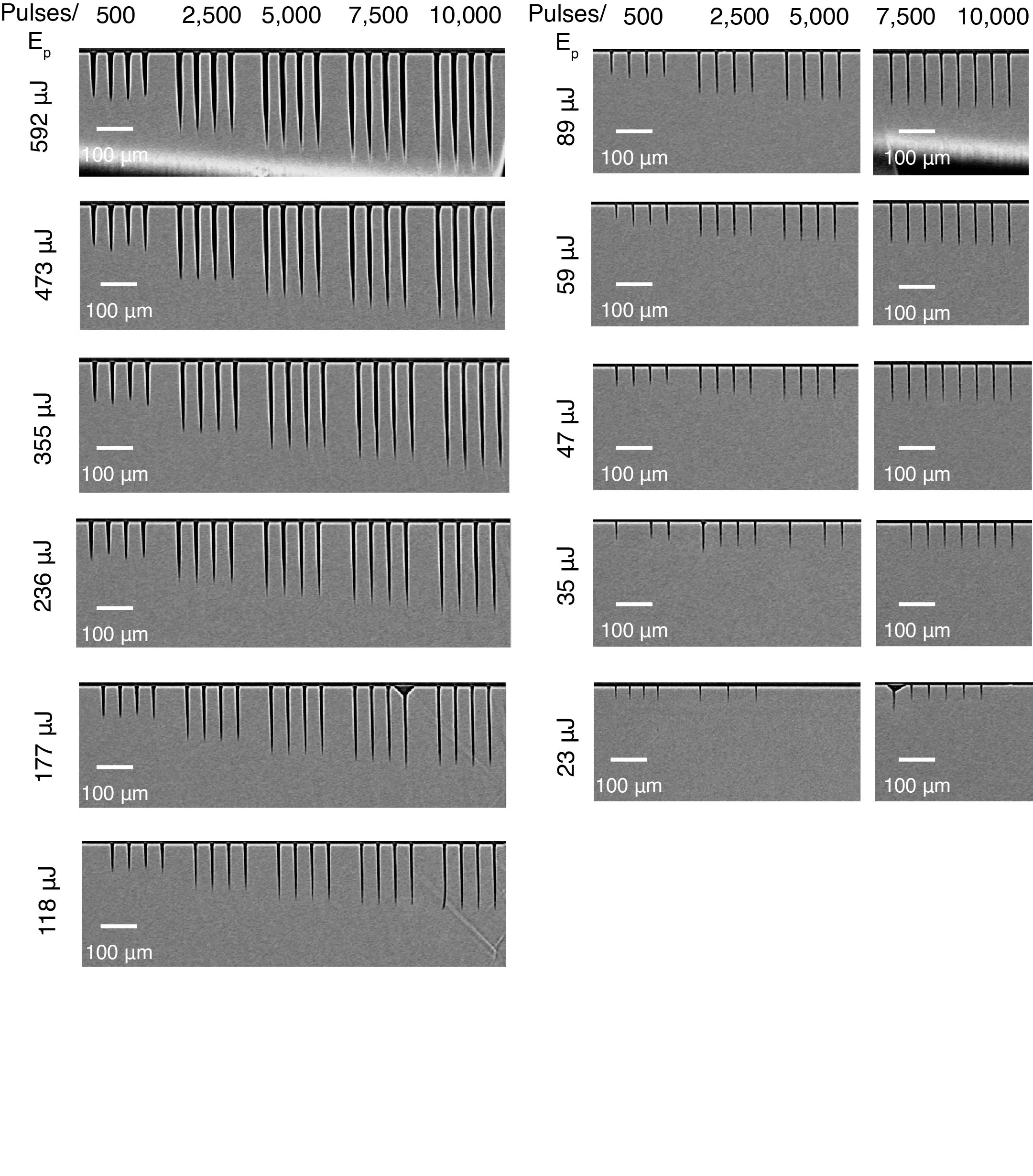


Figure S6. MicroCT Cross section views of percussion holes in the y-direction.

CT scans were acquired with 2.1 µm voxel resolution. Each image is an average of 3 adjacent slices to ensure the center of the hole is captured for each sample.


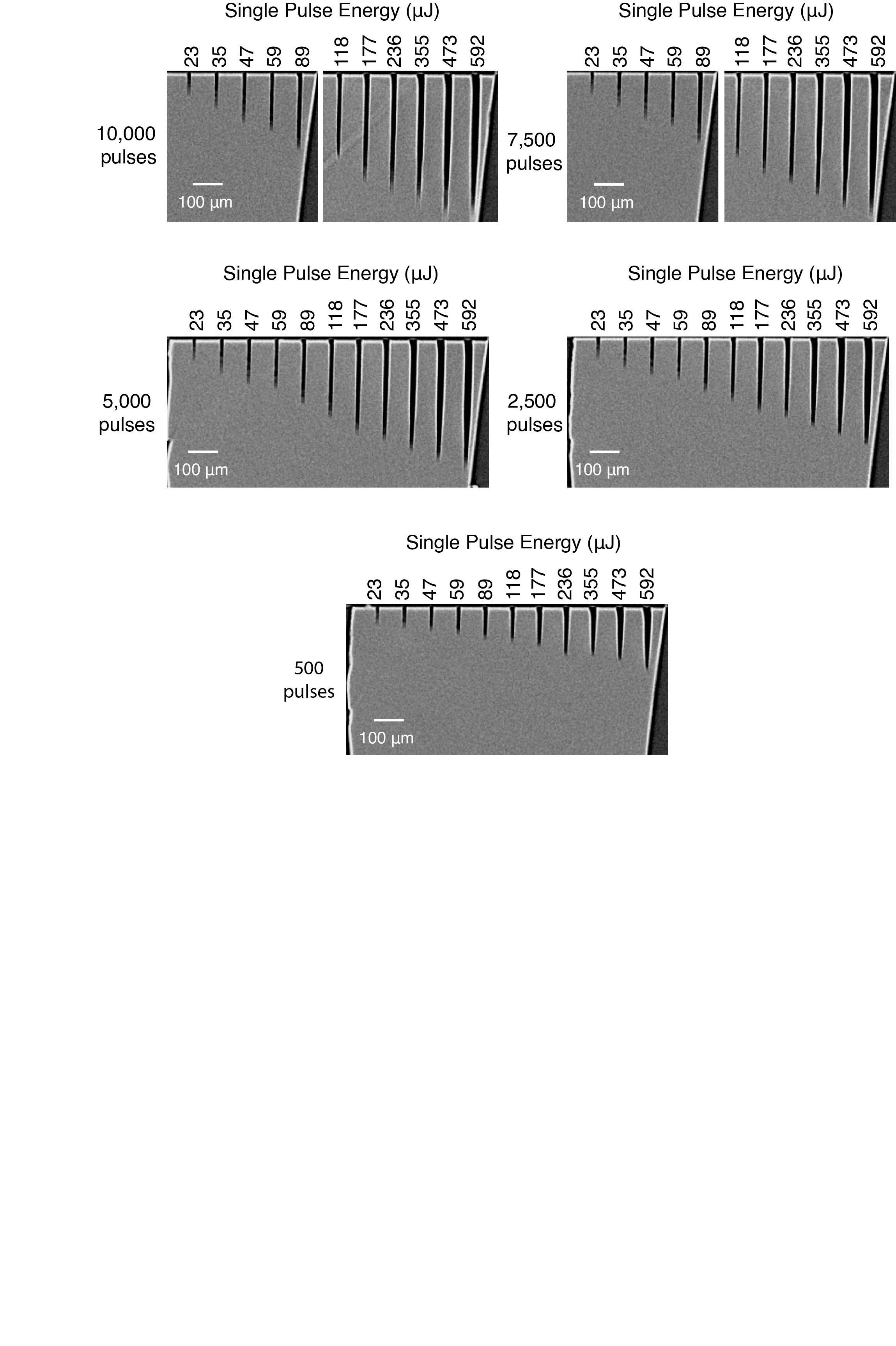
Figure S7. MicroCT cross section views of percussion holes in the x-direction.

CT scans were acquired with 2.1 µm voxel resolution. Each image is an average of 3 adjacent slices to ensure the center of the hole is captured for each sample.


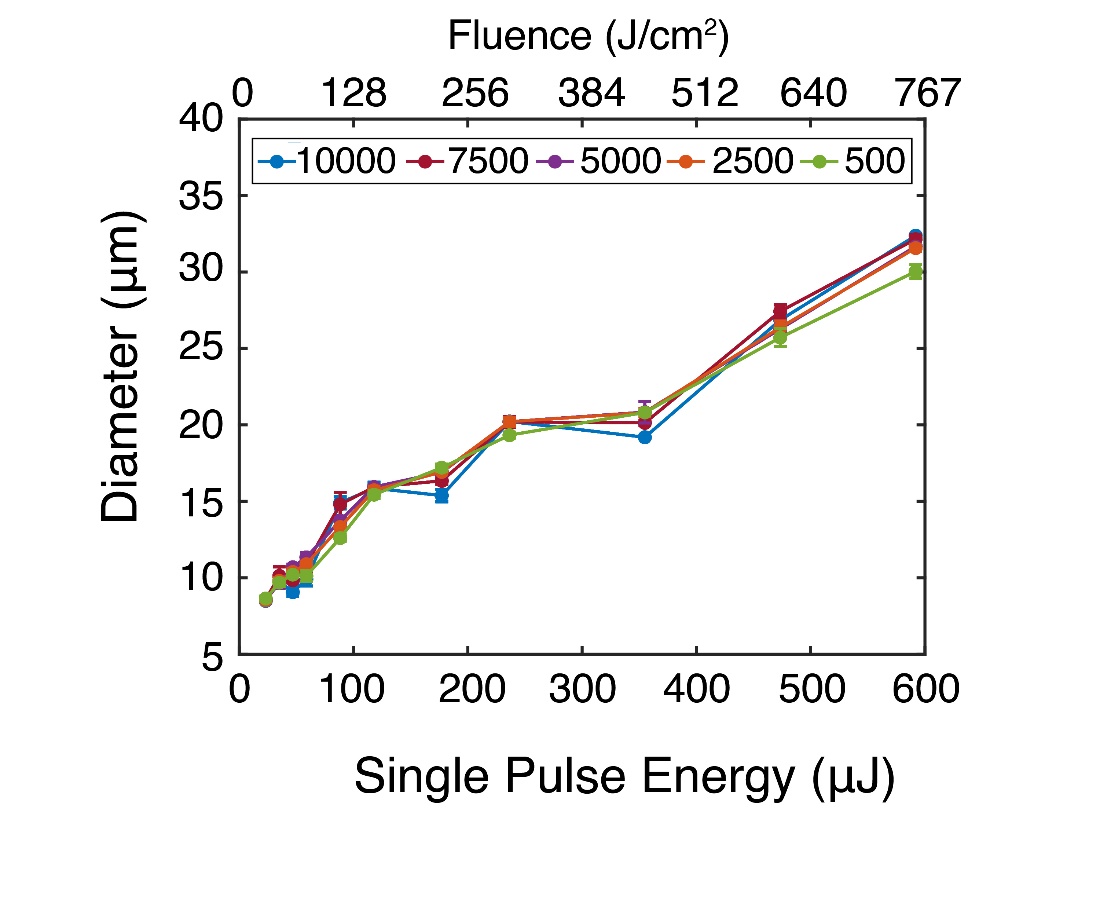


Figure S8. Inner diameter of percussion holes.

The inner diameter of percussion holes are shown for varied pulse energy and fluence, along with the number of pulses ranging from 10,000 to 500. The pulse diameter was variable on the order of the CT scanner resolution at 2.1 μm voxel resolution for a given pulse energy and varied pulse numbers. However, despite this variation, the overall trend describes the incubation effects shown in Figure SX and trend toward increased hole diameter with pulse energy.

| Pulse Number | Spot Size | Gentle Ablation single pulse energy threshold (µJ) | Gentle Ablation threshold fluence (J/cm^2^) | Strong Ablation single pulse energy threshold (µJ) | Strong Ablation threshold fluence (J/cm^2^) |
| --- | --- | --- | --- | --- | --- |
| 10,000 | 12.9 ± 1.1 | 9.6 ± 0.5 | 12.4 ± 1.4 | 124.7 ± 13.7 | 161.6 ± 22.4 |
| 7,500 | 13.7 ± 0.2 | 11.5 ± 2.8 | 14.9 ± 3.5 | 120.7 ± 18.9 | 156.4 ± 26.5 |
| 5,000 | 14.3 ± 0.3 | 12.6 ± 2.5 | 16.4 ± 3.2 | 109.1 ± 22.1 | 141.4 ± 28.7 |
| 2,500 | 14.7 ± 0.2 | 13.3 ± 1.8 | 17.2 ± 2.6 | 102.5 ± 26.6 | 132.7 ± 32.9 |
| 500 | 14.8 ± 0.1 | 13.2 ± 2.5 | 17.0 ± 3.3 | 97.9 ± 24.5 | 126.9 ± 30.5 |

Table S1. Ablation threshold of HPHT diamond.

The ablation threshold in gentle and strong regimes are provided as a function of pulse number. For all fluences, the average fluence was computed.


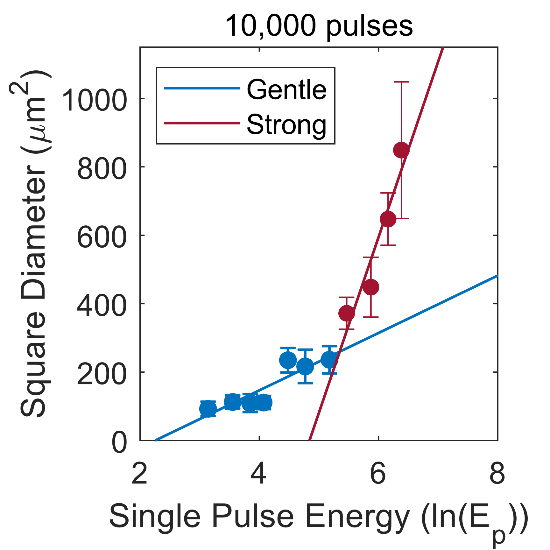

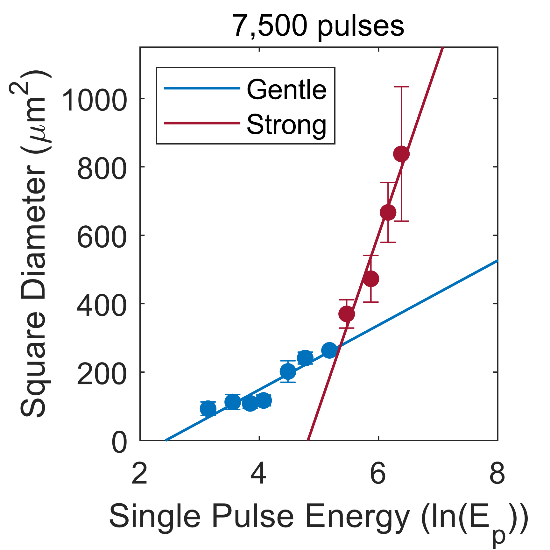


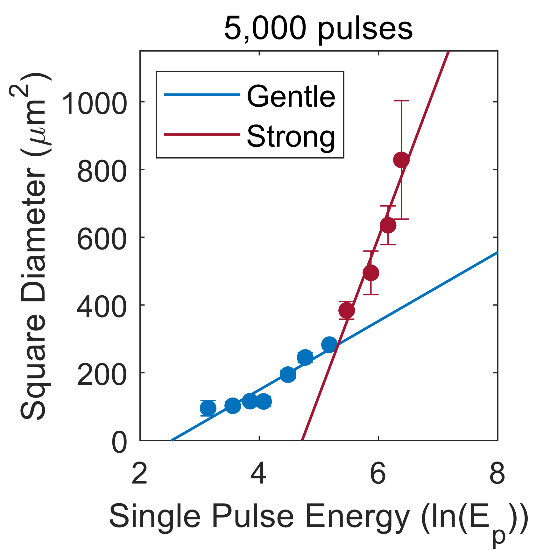

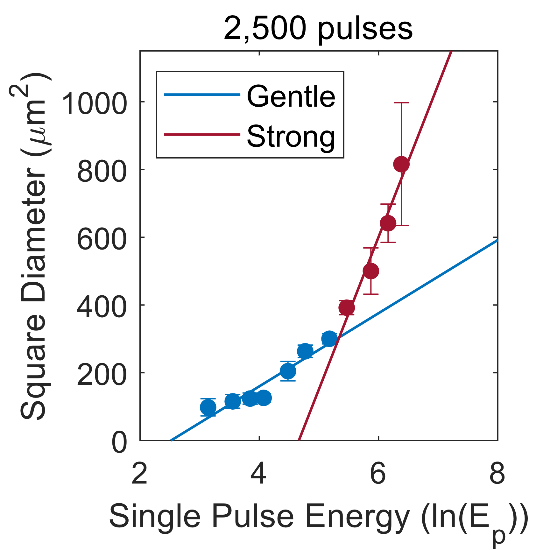

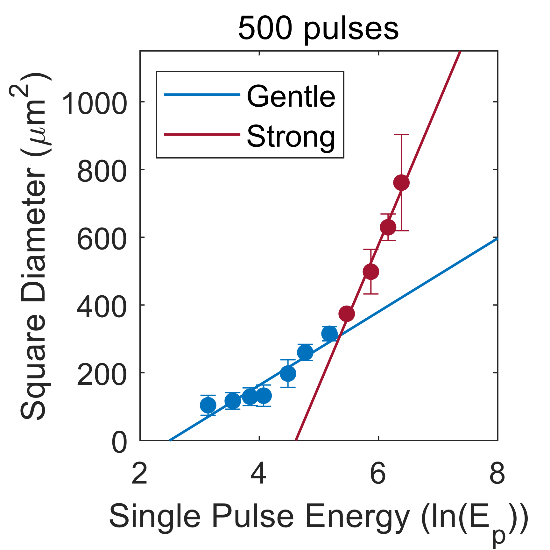


Figure S9. Determination of multiple pulse ablation threshold.

The effect of multiple pulses on the square of the hole diameter as a function of single pulse energy. The red plot indicates the strong ablation regime, while the blue plot is for the gentle ablation regime.


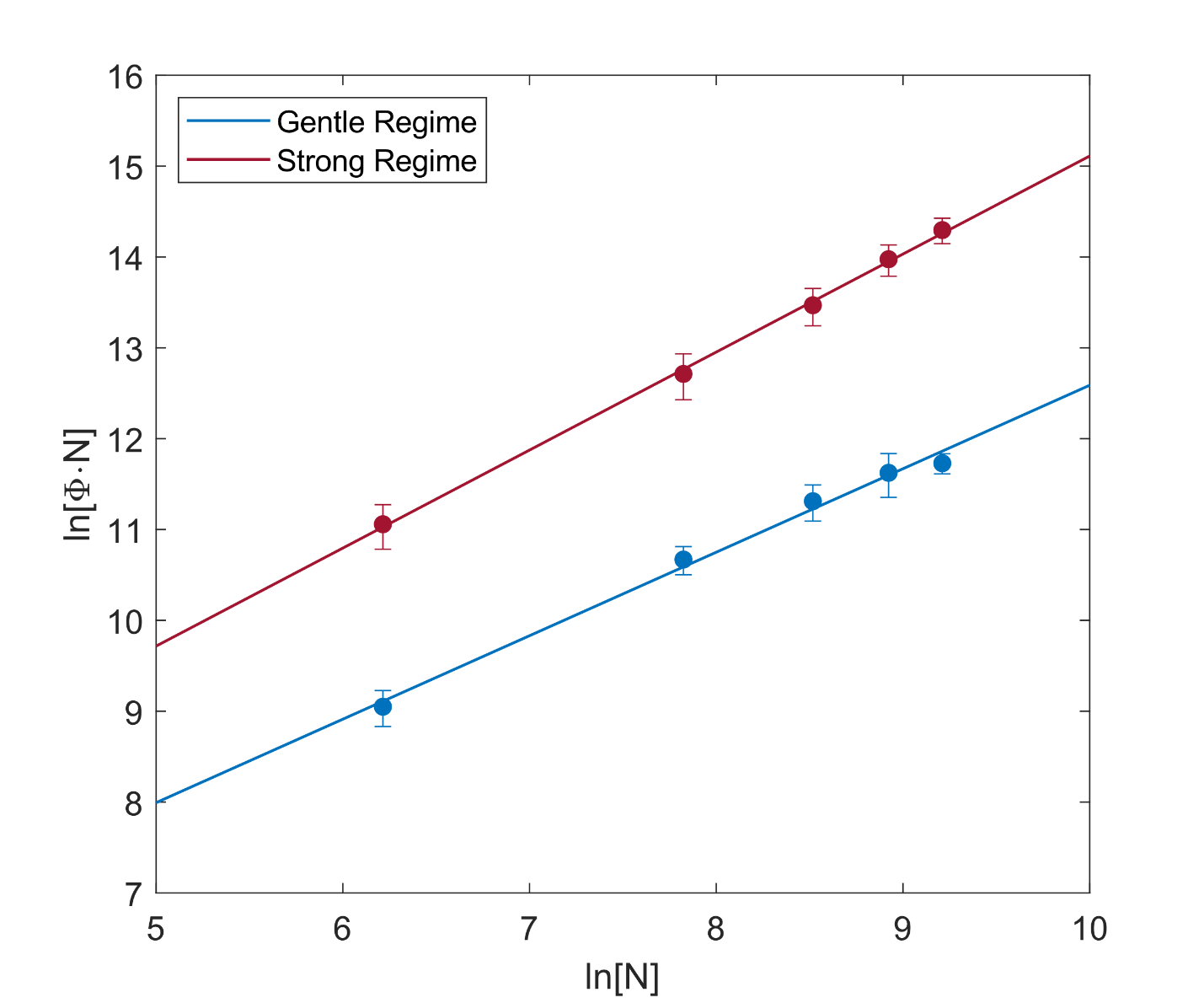


Figure S10. Single pulse ablation threshold.

The single pulse ablation threshold is determined from the plot of the natural log of the pulse number vs. the natural log of the product of the N-pulse ablation threshold, and the number of pulses, N.

**Section S3: ~ 40:1 Aspect Ratio Holes**


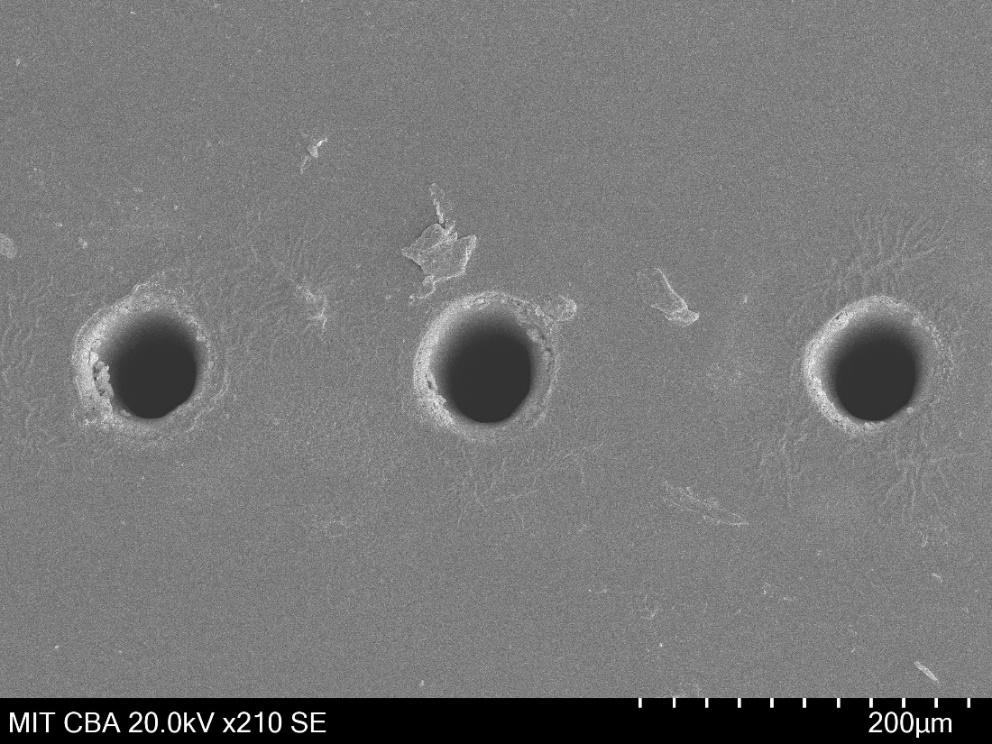


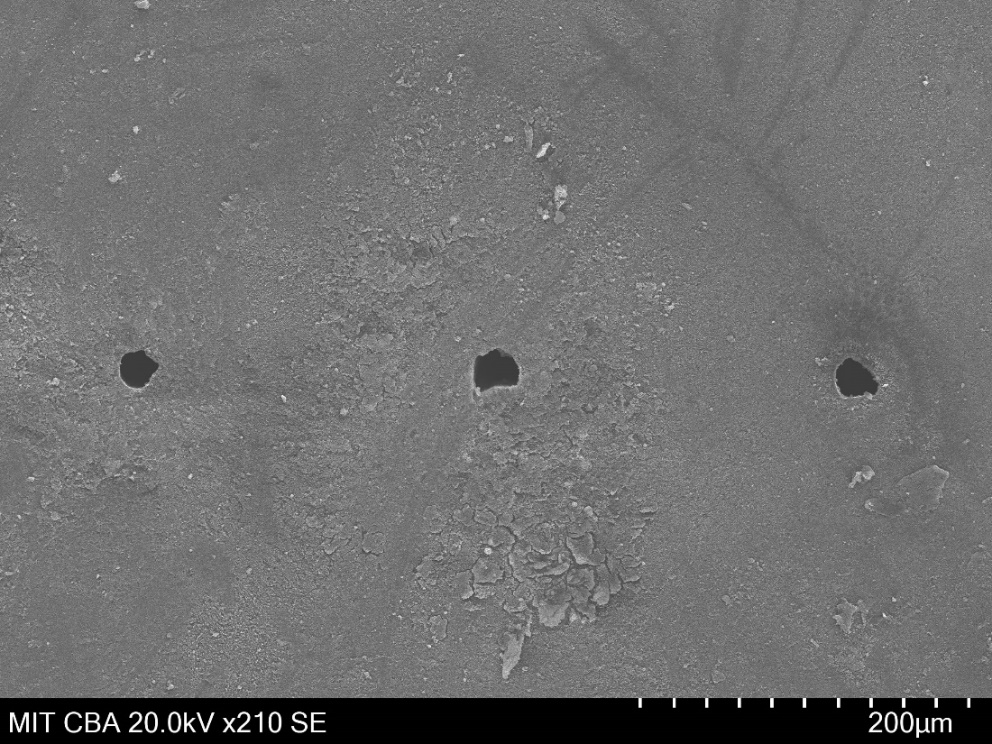


Figure S11. Entrance and Exit holes of ultra-high aspect ratio holes.

SEM of the entrance and exit hole sides of holes 1 through 3. Diamond sample was sputter coated with 100 nm of gold prior to SEM.

| Position (mm) | 0 | 0.4 | 0.67 | 1.3 | 1.6 | Average |
| --- | --- | --- | --- | --- | --- | --- |
| Diameter hole 1 (µm) | 79.7 | 46.4 | 29.7 | 42.6 | 24.1 | 44.5 |
| Diameter hole 2 (µm) | 77.9 | 46.3 | 33.4 | 40.8 | 31.6 | 46.0 |
| Diameter hole 3 (µm) | 79.8 | 46.4 | 31.6 | 42.7 | 29.7 | 46.0 |

Table 2. Inner diameters of 40:1 aspect ratio holes.

Hole inner diameter (µm) as a function of position down the depth of the hole for each of the three holes shown in Figure 2. A position of 0 mm corresponds to the surface of the entrance of the laser machined hole, while a position of 1.6 mm corresponds to the bottom surface of the exit hole.

| Position (mm) | 0 | 0.4 | 0.67 | 1.3 | 1.6 | Average |
| --- | --- | --- | --- | --- | --- | --- |
| Aspect ratio hole 1 | 20.1 | 34.5 | 53.9 | 37.6 | 66.4 | 42.5 |
| Aspect ratio hole 2 | 20.5 | 34.6 | 47.9 | 39.2 | 50.6 | 38.6 |
| Aspect ratio hole 3 | 20.1 | 34.5 | 50.6 | 37.5 | 53.9 | 39.3 |

Table 3. Aspect Ratio of ~40:1 holes.

Aspect ratio as a function of position down the depth of the hole for each of the three holes in Figure 2.


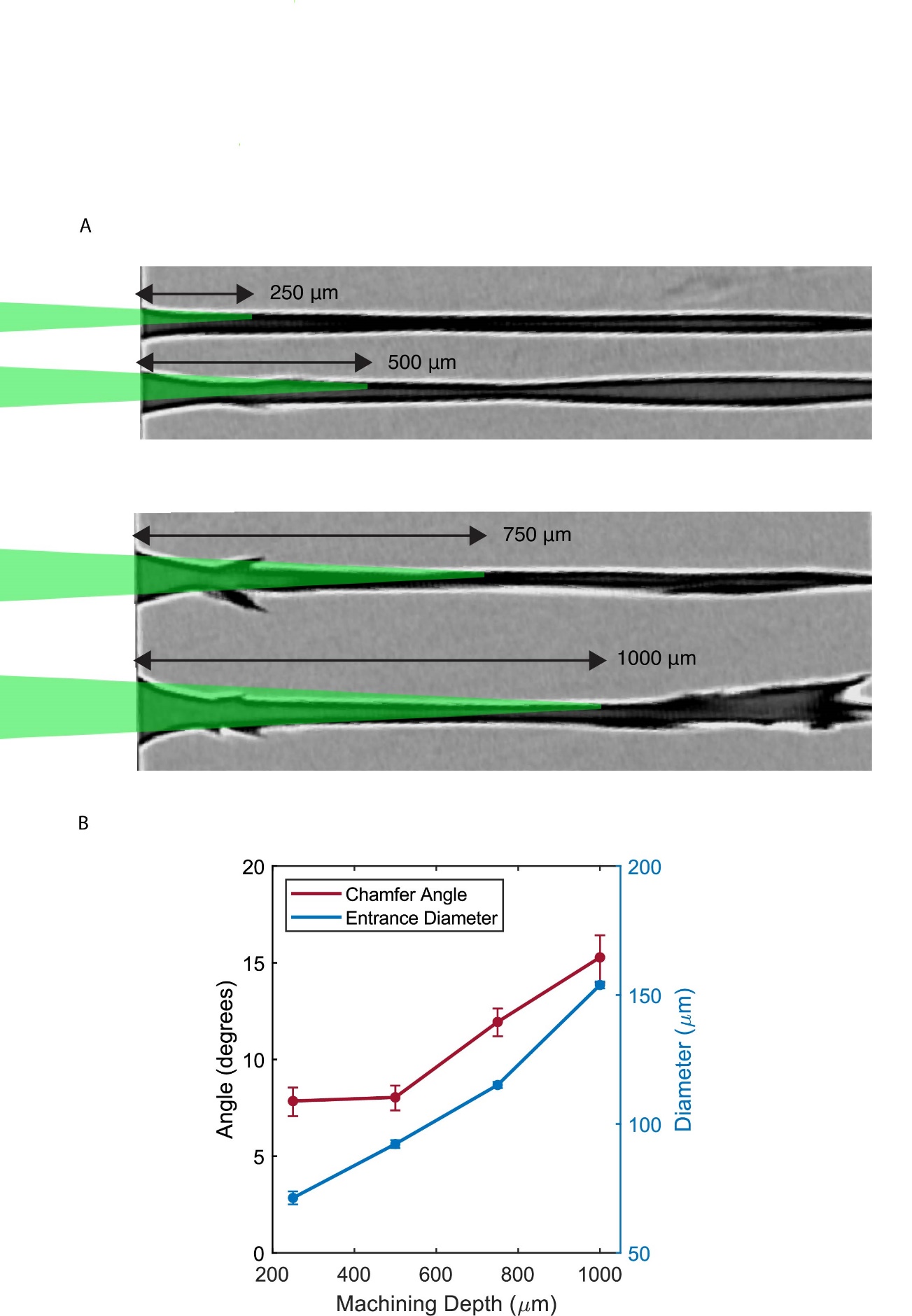


Figure S12. Chamfer as a function of machining depth.

The fabrication of ~40:1 high aspect ratio holes resulted in considerable observed chamfer near the top ~150 µm of the entrance hole. The laser beam profile is overlaid onto the hole in green and centered 10 µm from the center of rotation of the part, representing the 20 µm nominal hole diameter toolpathed. The observed chamfer increases linearly to the machining depth. The machining depth is defined as the distance the z-axis is stepped down to maintain optical focus into the material during machining.

**Section S4. 10:1 Aspect ratio tubes**


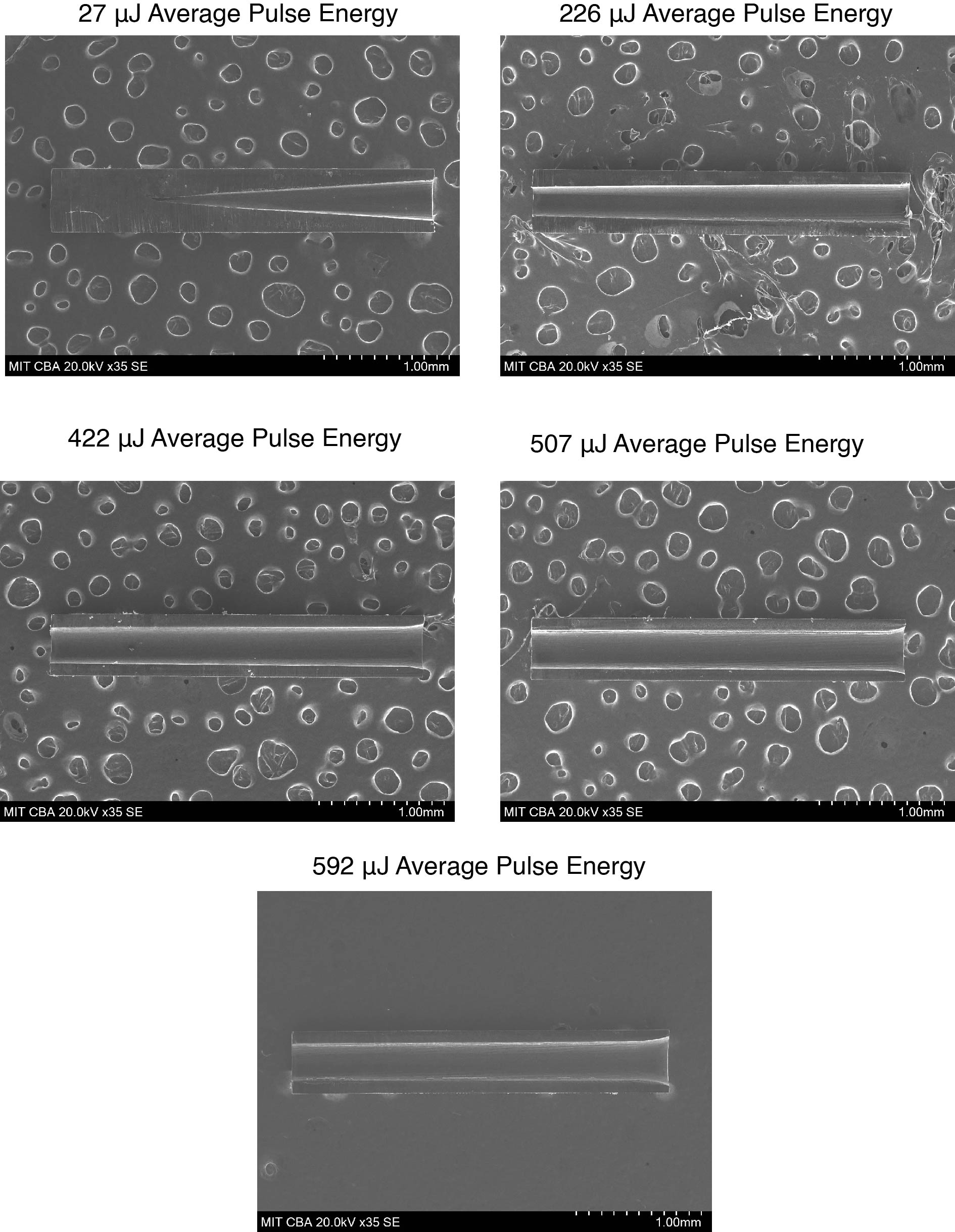


Figure S13. Uncropped SEMs of 10:1 Aspect Ratio tubes.

Uncropped images of the 10:1 aspect ratio tubes in Figure 3.


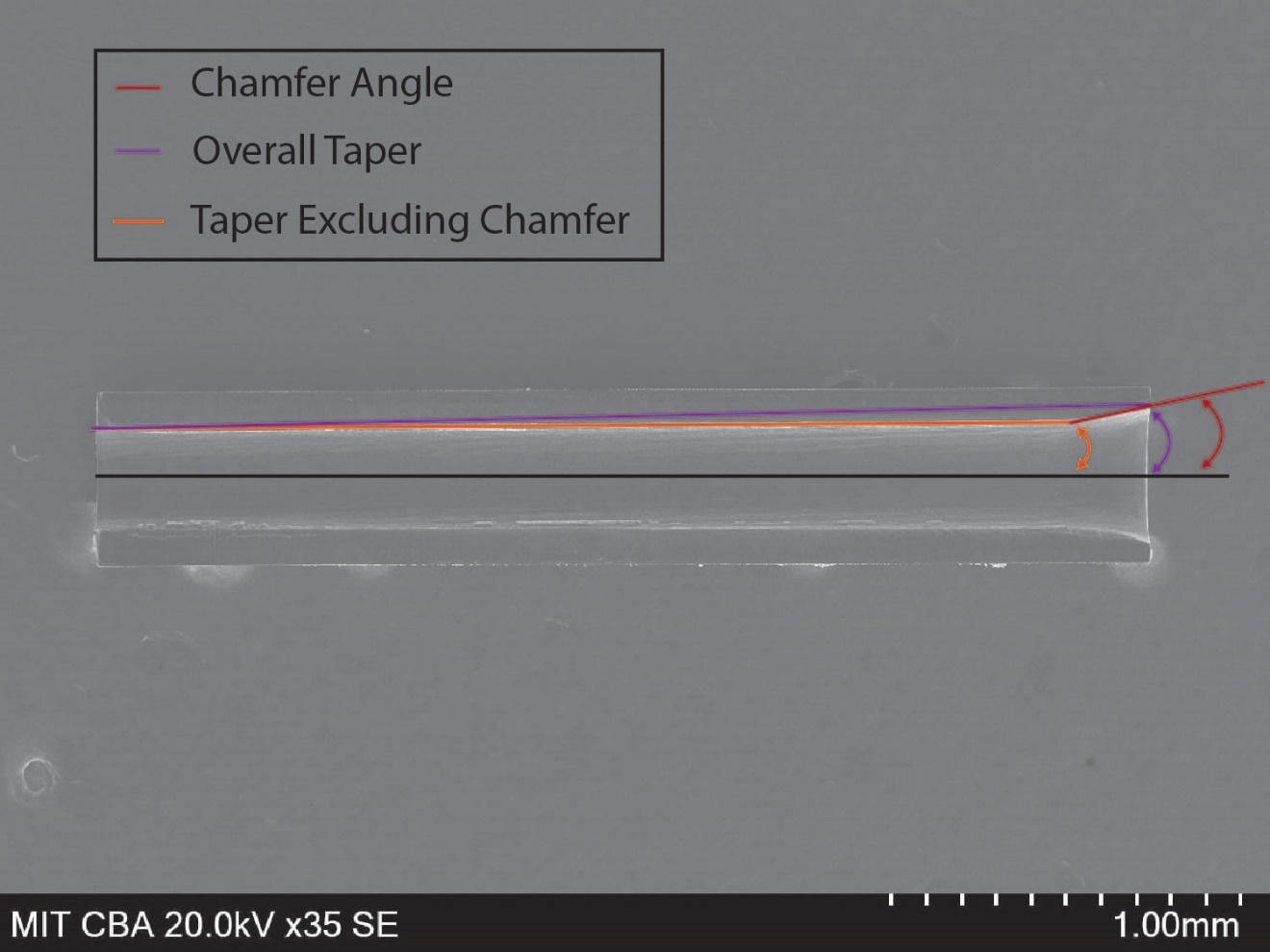


Figure S14. Definition of chamfer and taper angles.

Chamfer and taper angles were determined by analyzing SEM data using Autodesk Inventor CAD software.

| Average Single Pulse Energy  (µJ) | Chamfer Angle (Degrees) | Overall Taper  (Degrees) | Taper Excluding Chamfer (Degrees) | Entrance Diameter  (µm) |
| --- | --- | --- | --- | --- |
| 27 | 4.03 ± 0.04 | 4.03 ± 0.04 | 4.03 ± 0.04 | 325.0 ± 1.7 |
| 226 | 10.55 ± 2.01 | 0.92 ± 0.05 | 0.69 ± 0.05 | 328.7 ± 4.2 |
| 422 | 11.83 ± 0.51 | 0.80 ± 0.02 | 0.33 ± 0.04 | 371.2 ± 2.0 |
| 507 | 16.27 ± 1.14 | 0.72 ± 0.04 | 0.11 ± 0.05 | 388.9 ± 0.8 |
| 592 | 16.80 ± 1.50 | 0.95 ± 0.06 | 0.17 ± 0.05 | 399.2 ± 0.8 |

Table 4. Chamfer, taper, and diameter of 10:1 aspect ratio tubes.


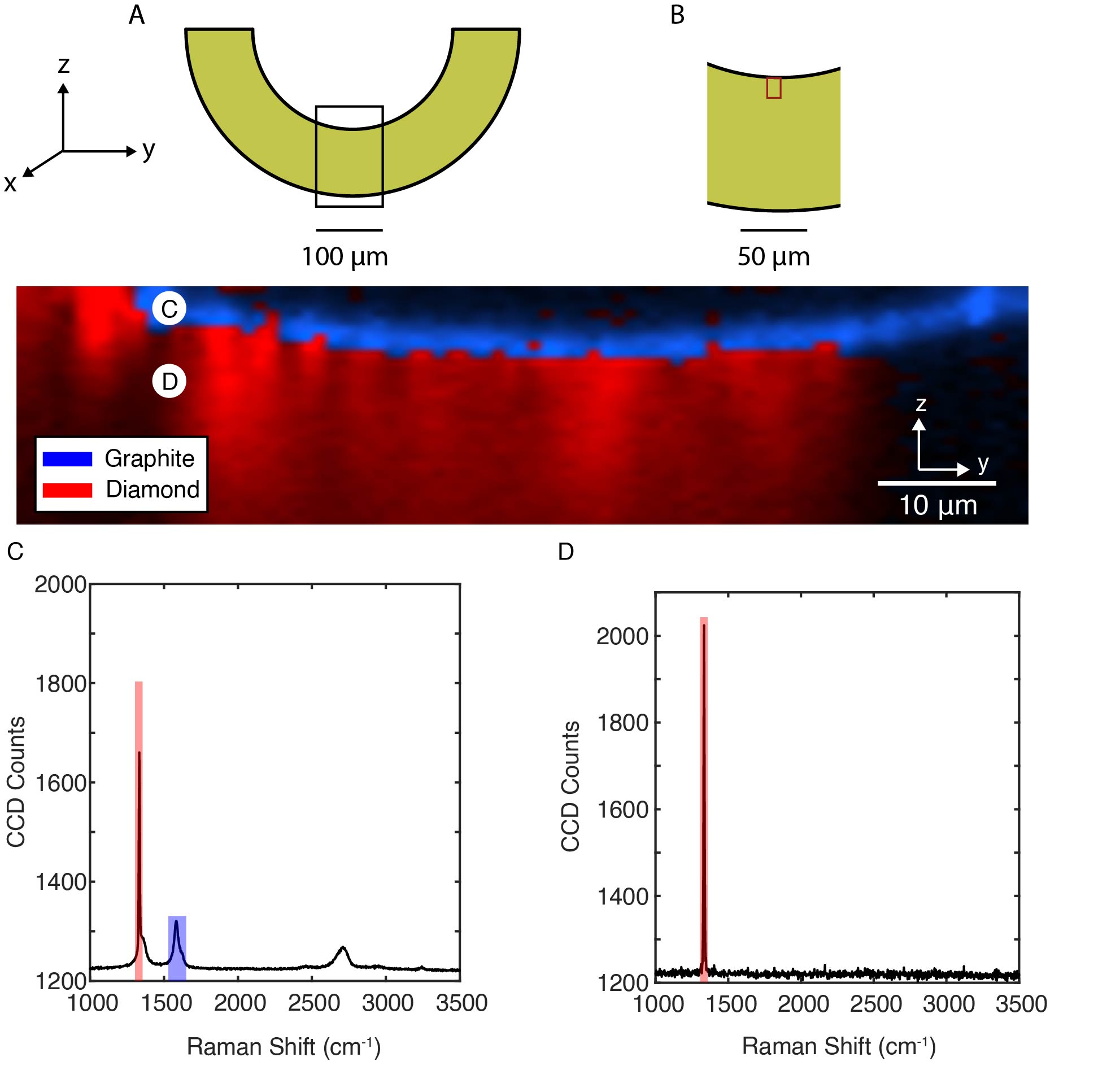


Figure S15. Surface Graphitization during ns laser machining.

Under nanosecond laser machining of diamond, laser absorption drives a phase transition from diamond to graphite. While most graphite is vaporized during machining, a thin layer, ~ 2 µm, remains on the machined inner surface. In the figure, the graphite signal is colored blue and corresponds to the sum of signals originating from the Raman shift of 1594 ± 60 cm^-1^. The diamond signal is colored red and corresponds to the sum of signals originating from the Raman shift of 1332 ± 25 cm^-1^.


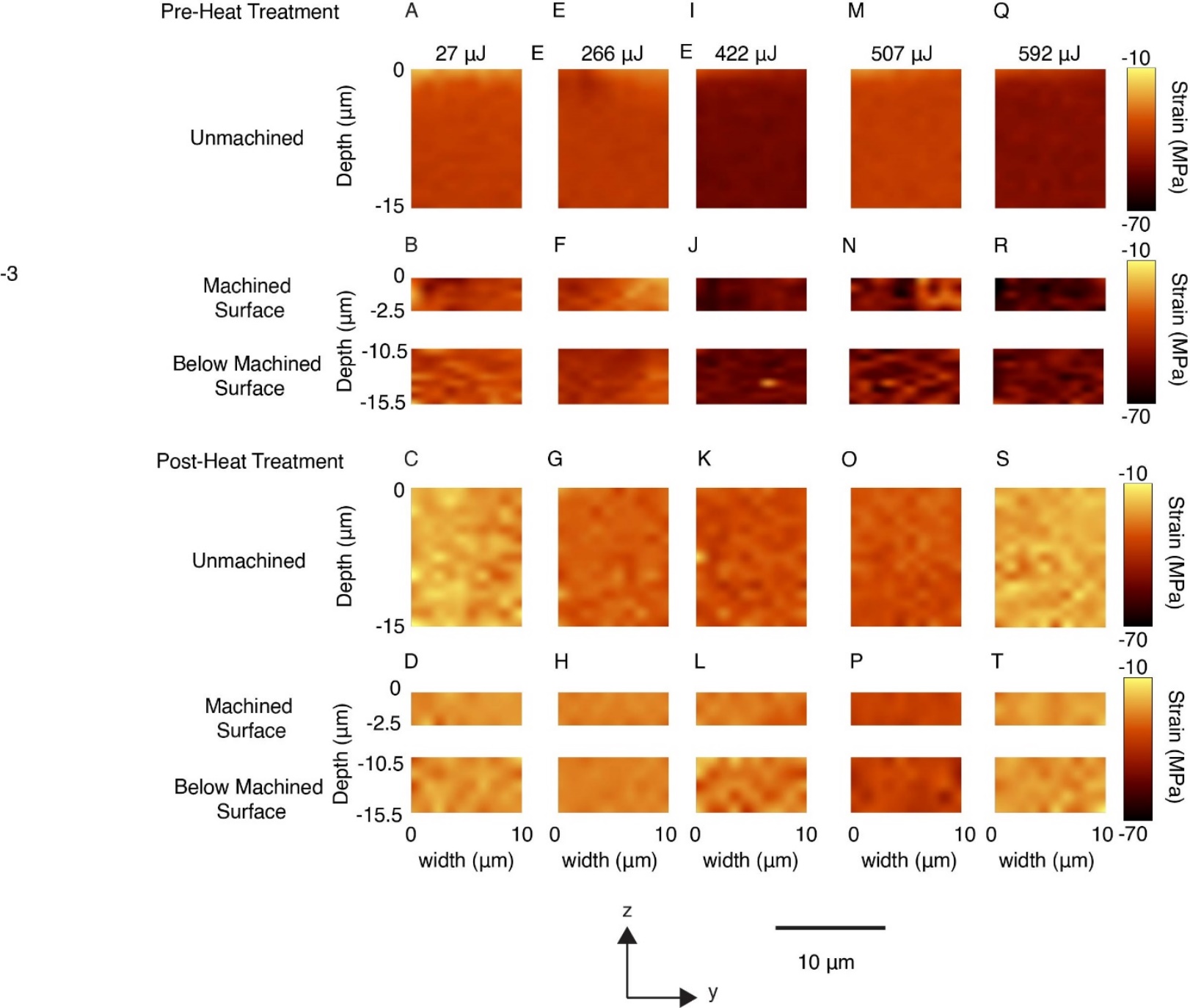


Figure S16. Raman images of 10:1 aspect ratio tube cross sectional samples.

Each image is 10 µm wide and the result of 900 accumulations averaged across three locations on the sample at 250 µm, 1250 µm, and 2750 µm down the tube length with an integration time of 0.2s. No significant changes were observed as a function of location along the tube. Between each strain measurement, the Raman shift of the spectrometer internal reference diamond was obtained and used to account for any spectrometer instabilities. The Raman shift distribution in the sample was determined using a Lorentzian fit in WITec Project software. For the unmachined reference samples, a depth of 15 µm was profiled from the top of the sample. For surface strain measurements, a depth of -1 ± 2 µm was profiled, while for the below surface strain measurements a depth of -13 ± 2 µm was profiled.


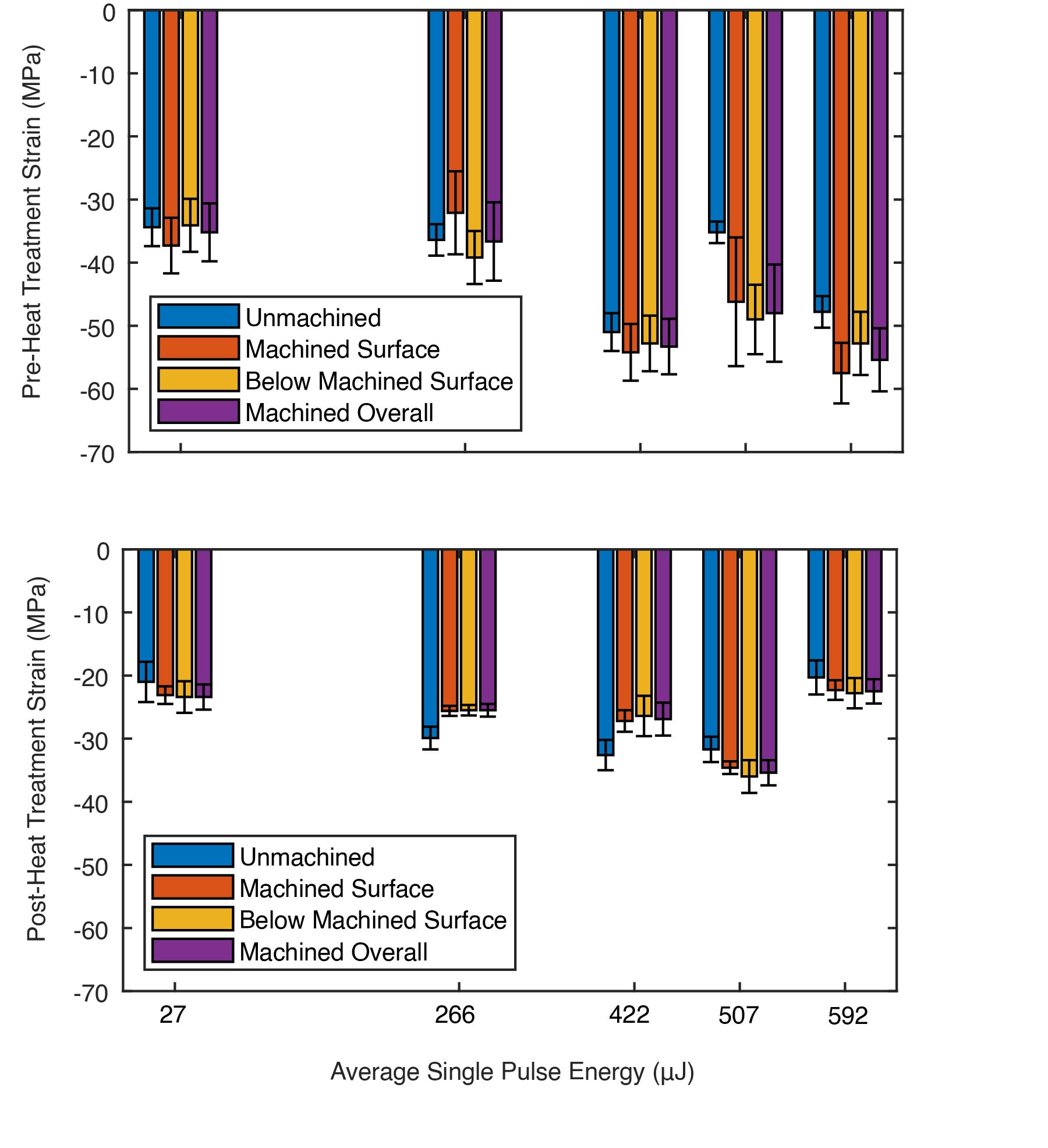


Figure S17. Pre and Post Heat Treatment Strain.

Observed strain in the diamond crystal as a function of machining power before and after heat treatment at 600° C for 24 hours. In all cases, high temperature treatment reduces the observed internal strain in HPHT diamond. This step importantly mitigates any strain introduced during the machining process.


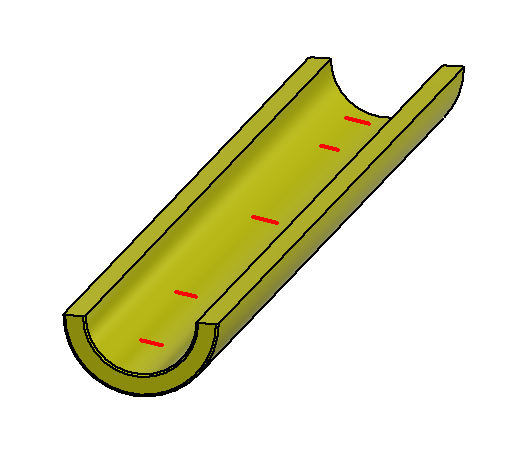

Supplement: Supplementary file 1 — Supplementary file1 (DOCX 5544 KB) [file 339_2023_6755_MOESM1_ESM.docx]
